# A Parallel Plate Variable Capacitor-Based Wind Pressure Sensor: Closed-Form Solution and Numerical Design and Calibration

**DOI:** 10.3390/s25123760

**Published:** 2025-06-16

**Authors:** Xiao-Ting He, Jun-Song Ran, Jing-Miao Yin, Jun-Yi Sun, Ying Guo

**Affiliations:** 1 School of Civil Engineering, Chongqing University, Chongqing 400045, China; hexiaoting@cqu.edu.cn (X.-T.H.); 202316131371@stu.cqu.edu.cn (J.-S.R.); 202416021167t@stu.cqu.edu.cn (J.-M.Y.); sunjunyi@cqu.edu.cn (J.-Y.S.); 2 State Key Laboratory of Safety and Resilience of Civil Engineering in Mountain Area, Chongqing 400045, China; 3 Key Laboratory of New Technology for Construction of Cities in Mountain Area of Ministry of Education (Chongqing University), Chongqing 400045, China

**Keywords:** wind pressure measurement, capacitive sensor, parallel plate variable capacitor, closed-form solution, numerical design, numerical calibration

## Abstract

In this paper, a parallel plate variable capacitor-based wind pressure sensor is proposed, which uses a wind-driven peripherally fixed circular membrane as its pressure-sensitive element and a spring-reset parallel plate variable capacitor as its sensing element. The circular membrane is first driven by the wind, and then it pushes the spring-reset movable electrode plate of the parallel plate variable capacitor to move, resulting in a change in the capacitance of the capacitor. The wind pressure, i.e., the direct action force per unit area exerted by the wind on the circular membrane, is thus detected by measuring the capacitance change of the capacitor. The elastic contact problem between the circular membrane and the spring-reset movable electrode plate is analytically solved, and its closed-form solution is presented, where the usually adopted small rotation angle assumption of the membrane is given up. The analytical relationship between the input pressure and output capacitance of the capacitive wind pressure sensor proposed here is derived. The validity of the closed-form solution is proved, and how to use the closed-form solution and input/output analytical relationship for the numerical design and calibration of the capacitive wind pressure sensor proposed here is illustrated. Finally, the qualitative and quantitative effects of changing design parameters on the capacitance–pressure analytical relationship of the wind pressure measurement system are investigated comprehensively.

## 1. Introduction

The “wind” refers to the flow of the mixture of gases that surrounds the Earth on a large scale, that is, the bulk movement of air on the surface of the Earth, caused by differences in atmospheric pressure. If an atmospheric pressure difference between two areas exists, the air will move from the higher-pressure area to the lower-pressure area, resulting in the winds of various strengths. How fast the air is moving is often defined as the strength of the wind, which is called the wind speed [1,2,3,4,5]. But sometimes, it is also defined by the pressure per unit area exerted by the moving air on a stationary surface, which is called the wind pressure [6,7,8,9,10]. As an action force, the direction of wind pressure is always opposite to the direction from which the wind originates, i.e., it is always opposite to the wind direction that is indicated by a pivoting weather vane.

Wind is usually measured for obtaining wind speed and wind direction, although the measurement mechanism is based on both velocity and pressure. Velocity-based measurement devices include cup [11], vane [12], hot-wire [13,14], laser Doppler [15], ultrasonic [16,17], acoustic resonance [18] and ping-pong ball [19] anemometers. The first designs of pressure-based anemometers were divided into plate and tube classes. Plate pressure anemometers are the first modern anemometers that were designed based on mechanical mechanisms [20,21], which usually use a square or circular flat plate normal to the wind to receive the action force exerted by the wind on the surfaces of the plates. The action force of the wind is balanced by a spring behind the plates. The wind pressure per unit area is determined by measuring the compression of the spring, and then the corresponding wind speed can be estimated. Since plate pressure anemometers are not very accurate, they are usually used only to trigger high-wind alarms, for example, on bridges. Tube pressure anemometers are probably the most commonly used pressure-based anemometers [22,23,24,25,26], which use a pitot–static tube with two ports to measure the stagnation pressure and static pressure. They are usually used for determining the airspeed of an aircraft [27,28,29,30] or the water speed of a boat [31]. Pitot tubes are particularly suitable for measuring the flow velocity of liquid, air, or gas in tubing or in ducts in certain industrial applications [32,33,34,35,36], because in these applications, measurements by other types of anemometers would be difficult to carry out, but the pitot tube can be inserted into the ducts through a small hole.

Although pitot-static tubes can be used to measure the stagnation pressure parallel to the wind direction and the static pressure perpendicular to the wind direction to determine the dynamic pressure of the wind being measured, there are still some special measurements of wind that are more concerned with the direct action force per unit area exerted by the wind on a rigid surface [37,38,39,40,41], rather than the stagnation or static pressure or the dynamic pressure. This is somewhat similar to the case of using a plate pressure anemometer, but existing plate pressure anemometers do not meet the needs of these special measurements of wind (not only because of their poor accuracy, but also because they do not have the ability to address remote real-time automatic detection). Here, this direct action force per unit area exerted by the wind on a rigid surface is called the wind pressure, but it is different from the stagnation or static pressure measured by pitot tubes (the stagnation or static pressure is defined in terms of energy, since according to Bernoulli’s equation, their difference is equal to the dynamic pressure, the kinetic energy per unit volume of an incompressible fluid in motion, which is equal to half of the product of the fluid density and square of the fluid velocity).

The measurement of wind pressure considered here is mainly aimed at the measurements of the direct action force per unit area exerted by the wind on some slender structures or structural members, such as ultrahigh-rise buildings [42], long-span bridges [43], tall lifting equipment [44], wind-power towers [45], electric transmission towers [46], and tall light poles or wire poles [47]. These structures or structural members will experience cyclic stress or stress amplitude under the action of wind, so it is necessary to monitor the stress amplitude level and the cycle number of the cyclic stress at high stress levels to avoid wind-induced structural fatigue failure [48,49,50,51,52]. It is well known that the stress to be measured can generally be given only by strain gauges, so it is almost impossible to extensively detect wind-induced structural stress in real time. This is also the reason why the real-time monitoring of wind-induced structural fatigue failure should be implemented, but it is difficult to be implemented at present. On the other hand, the relationship between wind-induced structural stress and wind pressure can be established by analyzing the elastic behavior of structures under wind pressure. Since the action force exerted by wind on whole structures can be determined in real time by the measured values of wind pressure at a finite number of measuring points placed on the structures, based on the established relationship between wind-induced structural stress and wind pressure, the corresponding wind-induced structural stress can also be determined in real time. In other words, the real-time monitoring of wind-induced structural fatigue failure is feasible, as long as the wind pressure (the direct action force per unit area) at a finite number of measuring points can be obtained in real time. This means that the wind pressure measurement to be addressed here has potential application value.

In this study, to achieve the measurement of wind pressure (the direct action force per unit area), a parallel plate variable capacitor-based circular wind pressure sensor is proposed. It uses a wind-driven peripherally fixed circular membrane as a pressure-sensitive element to convert the wind pressure into membrane deflection, and it uses a spring-reset circular parallel plate variable capacitor as a sensing element to convert the membrane deflection into the capacitance of the capacitor. The circular parallel plate variable capacitor is composed of a pair of parallel circular electrode plates, one of which is not allowed to move, so it is called a fixed electrode plate, while the other one is allowed to move, so it is called a movable electrode plate. The movable electrode plate is pushed by the wind-driven peripherally fixed circular membrane to move, and when the driving force disappears, it is reset by a spring behind it (its behavior is somewhat similar to that of a circular plate pressure anemometer, so, from this point of view, the wind pressure sensor proposed here can also be regarded as being derived from an improvement on plate pressure anemometers). The circular membrane can play a role in protecting the capacitor from wind and rain, and it can be any elastic thin film with good resistance to sunlight and rain. In particular, the capacitance of the capacitor can be directly used as an electrical signal output. So, as long as the elastic behavior of the circular membrane under wind pressure can be analytically solved, the sensor proposed here is feasible.

The remainder if this paper is organized as follows. In next section, the structure and operating principle of the capacitive wind pressure sensor proposed here are introduced, and the important wind pressure–capacitance analytical relationship of the wind pressure sensor proposed here is derived, where the capacitance is a function of the maximum deflection of the peripherally fixed circular membrane being in contact elastically with the spring-reset movable electrode plate, while the wind pressure applied onto the circular membrane is included in the deflection function. The large deflection problem of the peripherally fixed circular membrane being in contact elastically with the spring-reset movable electrode plate is solved analytically, which is arranged in Appendix A to maintain the consistency of the article. A closed-form solution for the large deflection problem is presented in Appendix A, in which the deflection solution will be used for determining the important wind pressure–capacitance analytical relationship of the sensor to be designed, while the stress solution will be used for determining the geometric and physical parameters of the circular membrane (these parameters are essential for the selection of the circular membrane). In Section 3, the validity of the closed-form solution presented is proved, how to use the closed-form solution during the numerical design and calibration of the wind pressure sensor proposed here is illustrated, and the effect of changing design parameters on the input–output relationship of the wind pressure measurement system is comprehensively investigated. Concluding remarks are shown in Section 4.

In comparison with the existing capacitive sensors suitable for wind pressure measurement [8,9,10,41], the advantages of the capacitive wind pressure sensor proposed here are mainly reflected in the following aspects. In the sensor proposed in this paper, the movable electrode plate and the pressure-sensitive element are independent of each other, while in the sensors proposed in [8,9,10,41], the movable electrode plate and the pressure-sensitive element are not independent of each other. This results in the advantages that the independent pressure-sensitive element in the sensor proposed in this paper, i.e., the circular membrane, can play a role in protecting the capacitor from wind and rain, and it can be any elastic thin film with good resistance to sunlight and rain. The sensor proposed in this paper adopts a parallel plate variable capacitor with a larger area than the sensors proposed in [8,9], which makes the edge effect of parallel plate capacitors easier to be reduced. The sensors proposed in [10,41] adopt a non-parallel plate variable capacitor (making the precise calculation of the capacitance difficult) and adopt a conductive thin film as the pressure-sensing element and movable electrode plate (which needs a thin film with both good conductivity and good elasticity). However, as Dr. Ferran Reverter noted in his paper marking the 70th anniversary of the piezoresistive effect, “the most appropriate mechanical sensor for a given application is the one that better adapts to the technical requirements of that application” [53].

The wind pressure sensor proposed here is mainly used to measure the direct action of the wind on the exterior facade of structures and then achieve the real-time monitoring of wind-induced structural fatigue failure. Since such real-time monitoring needs to simultaneously use many such sensors to form a computer control system, the read-out electronic circuits of the used sensors are suitable for using the digital capacitance measurement circuits that can directly convert capacitance values into digital signals [54] (but due to the length of this article, it is not covered here).

## 2. Materials and Methods

The action of fast-moving air (wind) on the surface of an object is different from the action of static gas on the inner wall of its storage container. The direction of the action force of the static gas is always perpendicular to the surface being acted upon and changes with the shape of the surface, and thus the static gas pressure belongs to uniformly distributed normal loads. However, the action direction of the wind is always the direction of the moving air. So, as long as the direction of the fast-moving air does not change, the action direction of the wind does not change, and thus the wind pressure belongs to the uniformly distributed transverse loads. Obviously, transverse load detection is different from normal load detection; even if the same device is used, their detection mechanism or theory is different. The structure and operating principle of the wind pressure sensor proposed here are as follows.

The proposed capacitive wind pressure sensor consists of two key construction units. One of the two is a pressure-sensitive element, which uses a wind-driven peripherally fixed circular membrane to convert the wind pressure into the membrane deflection according to a certain law. The other one is a sensing element, which uses a spring-reset circular parallel plate variable capacitor to convert the membrane deflection into the capacitance of the capacitor according to a certain law.

**Figure 1:**
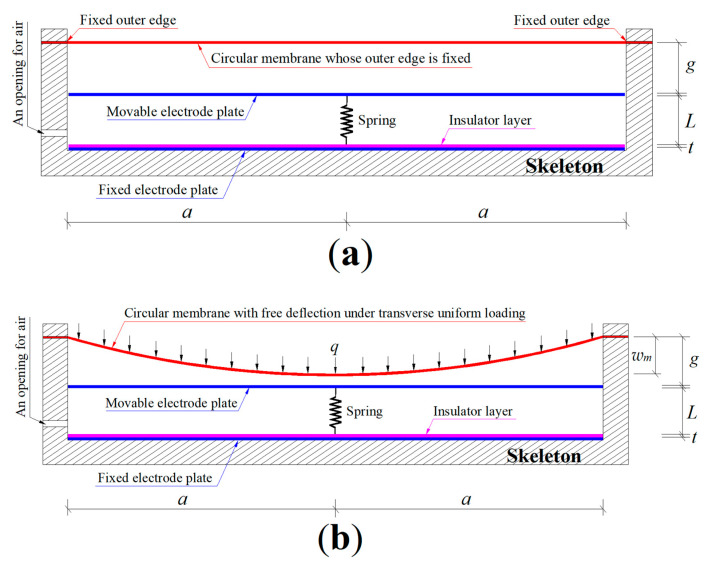

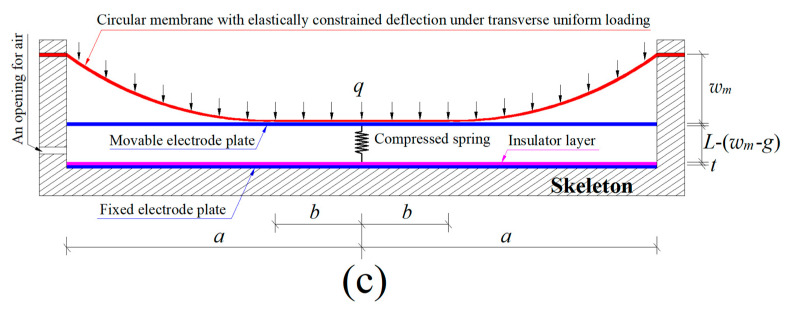
Sketch of the proposed wind pressure sensor: (**a**) the initial state without loading; (**b**) the situation before the circular membrane is in contact with the spring-reset movable electrode plate under the transverse uniform loading of the wind pressure *q*; (**c**) the situation after the circular membrane is in contact with the spring-reset movable electrode plate under the transverse uniform loading of the wind pressure *q*.

As shown in Figure 1a, a non-conductive cylindrical polymer tank with an inner radius *a* is used as a skeleton of the circular capacitive wind pressure sensor proposed here. The circular parallel plate variable capacitor is composed of a pair of parallel electrode plates, one of which is a fixed electrode plate and the other one is a movable electrode plate. The fixed electrode plate, a rigid conductive circular thin plate with a radius *a*, is first fixed to the bottom of the non-conductive cylindrical polymer tank and then coated by an insulator layer with a thickness *t* and a relative permittivity *ε_r_*_1_. The movable electrode plate is also a rigid conductive circular thin plate with a radius *a*, which is connected to the insulator layer by a spring with an original length *L*, where the stiffness coefficient of the spring is assumed to be denoted by *k* and the self-weight of the movable electrode plate is assumed to be able to compress the spring by Δ*l* (i.e., the self-weight of the movable electrode plate is equal to *k*Δ*l*). The circular membrane used as the pressure-sensitive element is initially flat (before it is subjected to the action of the wind pressure and its self-weight) and is fixed to the inner wall of the non-conductive cylindrical polymer tank, and thus its radius is the same as the inner radius *a* of the non-conductive cylindrical polymer tank. In addition, the circular membrane that is initially flat and peripherally fixed also needs to maintain a parallel gap *g* with the movable electrode plate that has not been moved yet (corresponding to the spring with the original length *L*), to be able to consider the membrane deflection due to the self-weight of the circular membrane, that is, to ensure that the circular membrane will not touch the movable electrode plate before being subjected to wind pressure.

Under the transverse uniform loading of the wind pressure *q*, as shown in Figure 1b, the circular membrane will deflect elastically toward the spring-reset movable electrode plate and produce a maximum deflection *w_m_*, but it has not yet been in contact with the spring-reset movable electrode plate, i.e., the maximum deflection *w_m_* is less than the initial parallel gap *g*, because at this time the wind pressure *q* is not yet large enough. The maximum deflection *w_m_* has included the deflection due to the self-weight of the circular membrane. This means that the initial parallel gap *g* should be greater than or equal to the deflection due to the self-weight of the circular membrane.

As the wind pressure *q* intensifies, this wind-driven circular membrane will be in contact with the spring-reset movable electrode plate, as shown in Figure 1c, where the contact radius between the wind-driven circular membrane and the spring-reset movable electrode plate is denoted by *b*. The movement of the spring-reset movable electrode plate is attributed to both its self-weight and the driving force from the wind-driven circular membrane, that is, at this time, the total spring compression Δ*L* (Δ*L* = *w_m_* − *g*) has included the initial spring compression Δ*l* due to the self-weight of the movable electrode plate. Therefore, at this time, the parallel gap between the spring-reset movable electrode plate and the insulator layer is equal to *L* − (*w_m_* − *g*), as seen in Figure 1c.

Therefore, after the wind-driven circular membrane is in contact with the spring-reset movable electrode plate, as shown in Figure 1c, the circular parallel plate variable capacitor between the movable and fixed electrode plates may be regarded as consisting of two series parallel plate capacitors with two different dielectric materials. One of the two dielectric materials is the insulator layer with thickness *t* and relative permittivity *ε_r_*_1_ and the other one is the air (the relative permittivity of the air is denoted by *ε_r_*_2_, and *ε_r_*_2_ = 1.00053) between the spring-reset movable electrode plate and the insulator layer (the air gap is equal to *L* − (*w_m_* − *g*); see Figure 1c). If the vacuum permittivity is denoted by *ε*_0_ (*ε*_0_ = 8.854 × 10^−3^ pF/mm), then the capacitance *C*_1_ of the insulator layer capacitor may be written as

(1) $$C_{1}=\frac{\varepsilon_{0}\varepsilon_{r1}\pi a^{2}}{t}.$$

Also, the capacitance *C*_2_ of the air capacitor may be written as

(2) $$C_{2}=\frac{\varepsilon_{0}\varepsilon_{r2}\pi a^{2}}{L-w_{m}+g}.$$

The relationship between the total capacitance *C* of the sensor and the capacitances *C*_1_ and *C*_2_ is given by

(3) $$\frac{1}{C}=\frac{1}{C_{1}}+\frac{1}{C_{2}}.$$

From Equations (1)–(3), the total capacitance *C* of the sensor may finally be written as

(4) $$C=\frac{C_{1}C_{2}}{C_{1}+C_{2}}=\frac{\varepsilon_{0}\varepsilon_{r1}\varepsilon_{r2}\pi a^{2}}{\varepsilon_{r1}(L-w_{m}+g)+\varepsilon_{r2}t}.$$

It can be seen from Equation (4) that the capacitance *C* and the maximum deflection *w_m_* are in one-to-one correspondence. Therefore, the value of the maximum deflection *w_m_* can be determined from the measured value of the capacitance *C* through Equation (4). On the other hand, under the working condition in Figure 1c, the value of the maximum deflection *w_m_* of the circular membrane depends on the driving force of the wind pressure *q* and the restoring force of the compressed spring. Obviously, after the spring and the movable electrode plate are given, the spring stiffness coefficient *k* is constant and the initial spring compression Δ*l* is also constant (the self-weight of the movable electrode plate is fixed); only the wind pressure *q* is a variable. Therefore, the wind pressure *q* and the maximum deflection *w_m_* are also in one-to-one correspondence. This means that the capacitance *C* and the wind pressure *q* are in one-to-one correspondence. Therefore, as long as the relationship of the one-to-one correspondence between the capacitance *C* and the wind pressure *q* is known, the value of the wind pressure *q* can be determined by the measured value of the capacitance *C*; that is, the proposed wind pressure sensor can be realized.

The one-to-one corresponding relationship between the capacitance *C* and the wind pressure *q*, i.e., the analytical relationship between *C* and *q*, can only be determined by analytically solving the large deflection problem of the wind-driven peripherally fixed circular membrane being in contact elastically with the spring-reset movable electrode plate (see Figure 1c), which will be detailed in Appendix A.

## 3. Results and Discussion

In this section, some important issues will be addressed, which are critical to the numerical design and calibration of the sensor proposed here. As mentioned above, the closed-form solution derived in Appendix A plays a very important role in the development of the proposed wind pressure sensor. So, it should be discussed first whether there are derivation mistakes in the analytical solution to the membrane/plate elastic contact problem in Appendix A and whether the obtained closed-form solution is correct and valid, which will be detailed in Section 3.1. And then, how to use the closed-form solution derived in Appendix A for numerically designing and calibrating the proposed sensor is illustrated, which will be detailed in Section 3.2. Finally, the effect of changing design parameters on the input–output relationship of the sensor is comprehensively investigated, which is very important for the numerical design and calibration of the wind pressure sensor proposed here and will be detailed in Section 3.3.

### 3.1. Validity of Closed-Form Solution

**Figure 2:**
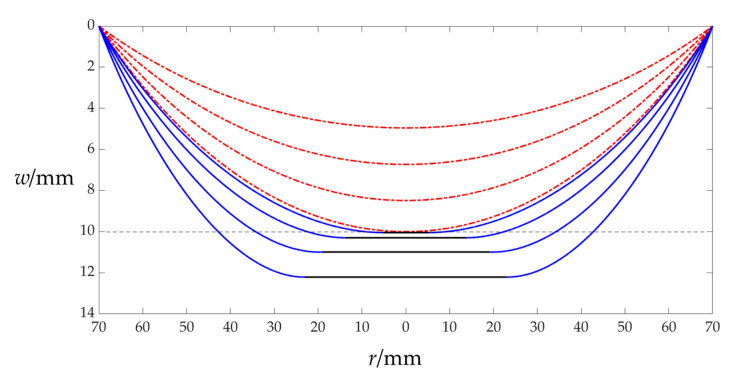
Sketch of the deflection curve in the case of membrane/plate contact (solid lines) gradually approaching the deflection curve in the case of membrane/plate non-contact (dot–dashed lines) with the decrease in the transverse uniform loads *q* acting upon the membrane, where the deflection curves (in bottom-up order) correspond to *q* = 650 Pa, *q* = 450 Pa, *q* = 250 Pa, *q* = 180 Pa, *q* = 163 Pa, *q* = 100 Pa, *q* = 50 Pa, and *q* = 20 Pa, respectively.

**Figure A1:**
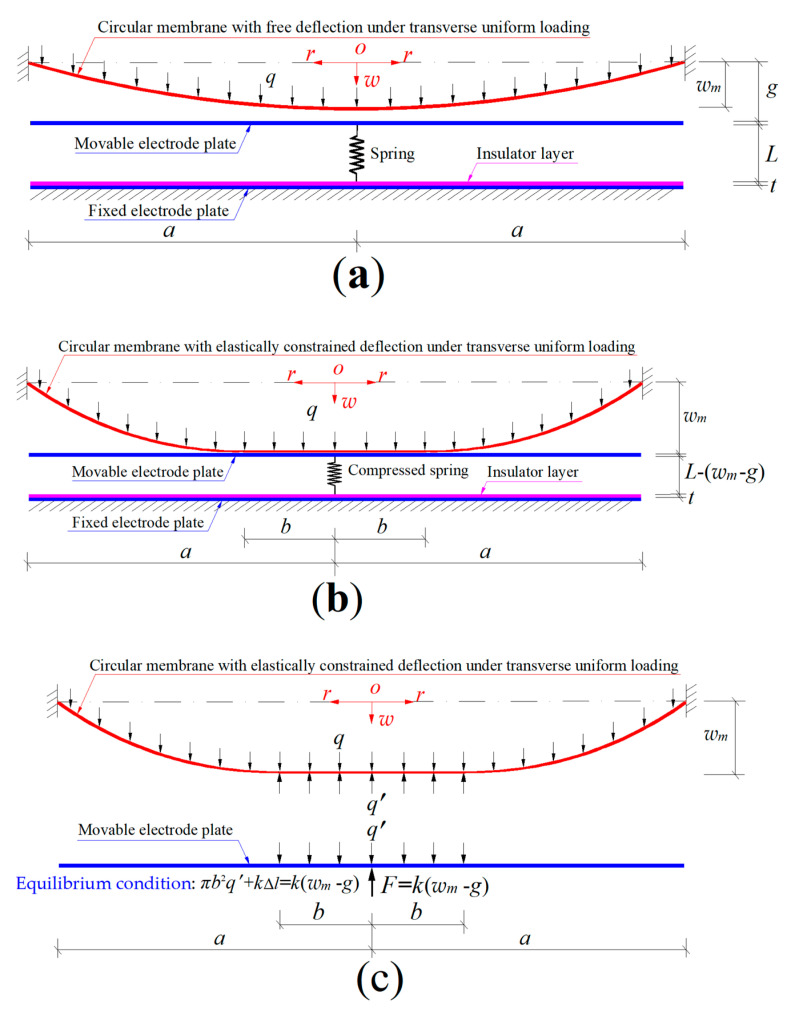
Sketch of the elastic behavior from non-contact to contact between the wind-driven circular membrane and the spring-reset movable electrode plate: (**a**) the membrane/plate non-contact state; (**b**) the membrane/plate contact state; (**c**) a decomposed view of the membrane/plate interaction force.

The closed-form solution of the membrane/plate elastic contact problem derived in Appendix A can be shown to be valid in the following way. Obviously, before the membrane/plate contact, as shown in Figure A1a, the deflection of the circular membrane under transverse uniform loads *q* is not constrained, while after membrane/plate contact, as shown in Figure A1b, the deflection of the circular membrane under transverse uniform loads *q* is constrained elastically by the spring-reset movable electrode plate, and within the membrane/plate contact region of radius *b*, the circular membrane is flat. In other words, the membrane/plate contact radius *b* will gradually decrease to zero as the transverse uniform loads *q* gradually decrease. Therefore, the deflection curve drawn with the membrane/plate contact closed-form solution (the closed-form solution obtained in Appendix A) should be continuous with the deflection curve drawn with the membrane/plate non-contact closed-form solution (the closed-form solution for the large deflection problem of the circular membrane in Figure A1a), that is, the deflection curve of the circular membrane under transverse uniform loads *q* should continuously change as the transverse uniform loads *q* gradually decrease. In other words, as the transverse uniform loads *q* gradually decrease, the deflection curve in the case of the membrane/plate contact should gradually approach or be close to the deflection curve in the case of membrane/plate non-contact. In this case, if the membrane/plate non-contact closed-form solution is valid, then the membrane/plate contact closed-form solution should also be valid; that is, the closed-form solution derived in Appendix A is valid. Figure 2 shown such a case of a gradual approach, where the four deflection curves in the case of membrane/plate contact (the four solid lines in Figure 2) are calculated using the closed-form solution derived in Appendix A, while the four deflection curves in the case of membrane/plate non-contact (the four dot–dashed lines in Figure 2) are calculated using a well-established closed-form solution for the large deflection problem of transversely uniformly loaded circular membranes presented in [55]. It can be concluded from Figure 2 that the closed-form solution derived in Appendix A should be valid.

The relevant geometric and physical parameters used in the deflection calculations for plotting Figure 2 are, respectively, radius *a* = 70 mm, thickness *h* = 0.3 mm, Poisson’s ratio *v* = 0.45, and Young’s modulus of elasticity *E* = 3.01 MPa for the used circular membrane, as well as an initial parallel gap *g* = 5 mm, a spring stiffness coefficient *k* = 0.5 N/mm, and an initial spring compression Δ*l* = 5 mm, while the transverse uniform loads *q*, respectively, take 20 Pa, 50 Pa, 100 Pa, 163 Pa, 180 Pa, 250 Pa, 450 Pa, and 650 Pa (in Figure 2, from top to bottom). When the transverse uniform loads *q* take 20 Pa, 50 Pa, 100 Pa, and 163 Pa, respectively, the undetermined constant *c* of the membrane/plate non-contact problem is *c* = 0.334131, *c* = 0.330840, *c* = 0.326663, and *c* = 0.322379, respectively, which is calculated using the closed-form solution presented in [55]. And when the transverse uniform loads *q* take 180 Pa, 250 Pa, 450 Pa, and 650 Pa, respectively, the undetermined constants *β*, *c*_0_, *c*_1–_, and *d*_0_ of the membrane/plate contact problem are *β* = 0.544050, *c*_0_ = 0.024999, *c*_1_ = −0.004715, and *d*_0_ = 0.107564 for *q* = 180 Pa, *β* = 0.598700, *c*_0_ = 0.029582, *c*_1_ = −0.005228, and *d*_0_ = 0.109212 for *q* = 250 Pa; *β* = 0.649548, *c*_0_ = 0.040977, *c*_1_ = −0.006322, and *d*_0_ = 0.119276 for *q* = 450 Pa; and *β* = 0.666143, *c*_0_ = 0.051272, *c*_1_ = −0.007420, and *d*_0_ = 0.129987 for *q* = 650 Pa, respectively, which are calculated using the closed-form solution derived in Appendix A.

**Figure 3:**
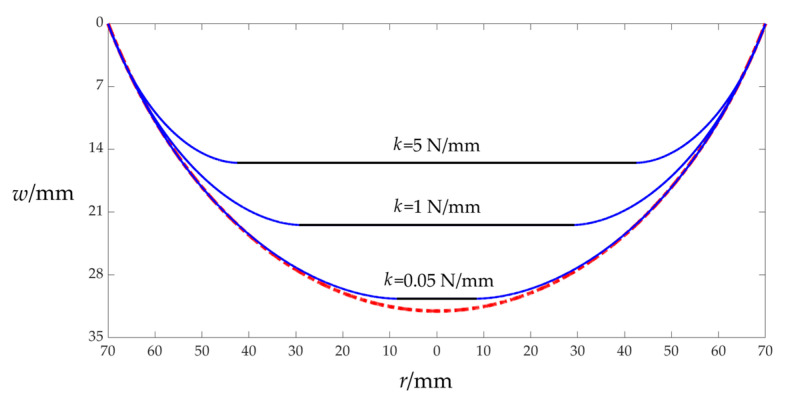
Sketch of the deflection curve with membrane/plate contact (solid lines) gradually approaching the deflection curve without membrane/plate contact (dot–dashed line) with the decrease in the spring stiffness coefficient *k* (reduced from 5 N/mm to 1 N/mm and then to 0.05 N/mm), where the transverse uniform loads *q* acting upon the same circular membrane are always kept to be *q* = 5000 Pa.

Similarly, the closed-form solution of the membrane/plate elastic contact problem derived in Appendix A can also be shown to be valid in the following way. Under the premise that the transverse uniform loads *q* remains constant, if the spring stiffness coefficient *k* is gradually reduced to zero, then the deflection curve in the case of membrane/plate contact will gradually approach the deflection curve in the case of membrane/plate contact (i.e., it is assumed that the same circular membrane will not be constrained elastically by the spring-reset movable electrode plate under the same transverse uniform loads *q*), as shown in Figure 3, where the three deflection curves in the case of membrane/plate contact (the three solid lines in Figure 3) are calculated using the closed-form solution derived in Appendix A, while the deflection curve of the same membrane under the same transverse uniform loads *q* (the dot–dashed line in Figure 3) is calculated using the closed-form solution presented in [55]. From Figure 3, it can also be concluded that the closed-form solution derived in Appendix A should be valid.

The relevant geometric and physical parameters used in the deflection calculations for plotting Figure 3 are the same as those for plotting Figure 2, but the spring stiffness coefficient *k* takes 5 N/mm, 1 N/mm, and 0.05 N/mm, respectively, and the transverse uniform loads *q* are always kept at 5000 Pa. In addition, the undetermined constants *β*, *c*_0_, *c*_1_, and *d*_0_ used to determine the three solid lines in Figure 3 are *β* = 0.799273, *c*_0_ = 0.155824, *c*_1_ = −0.011412, and *d*_0_ = 0.173902 for *k* = 5 N/mm; *β* = 0.701074, *c*_0_ = 0.212254, *c*_1_ = −0.020493, and *d*_0_ = 0.249306 for *k* = 1 N/mm; and *β* = 0.557908, *c*_0_ = 0.263520, *c*_1_ = −0.034924, and *d*_0_ = 0.341315 for *k* = 0.05 N/mm, respectively, which are calculated using the closed-form solution derived in Appendix A. Meanwhile, the undetermined constant *c* used to determine the dot–dashed line in Figure 3 is *c* = 0.217788, which is calculated using the closed-form solution presented in [55].

### 3.2. How to Use Closed-Form Solution for Numerical Design and Calibration

In this section, we will show how to use the closed-form solution derived in Appendix A to carry out the numerical design and calibration of the proposed wind pressure sensor.

The design parameters of the sensor to be designed include the following geometric and physical parameters. The geometric parameters include the inner radius *a* of the non-conductive cylindrical polymer tank (the radius of the circular membrane or movable and fixed electrode plates, see Figure 1), the original length *L* of the spring, the initial parallel gap *g* between the initially flat circular membrane and the movable electrode plate, the insulator layer thickness *t*, the membrane thickness *h*, and the initial spring compression Δ*l*. The physical parameters include the Young’s modulus of elasticity *E* and Poisson’s ratio *v* of the circular membrane, the spring stiffness coefficient *k*, and the relative permittivity *ε_r_*_1_ of the insulator layer. In addition, the vacuum permittivity is *ε*_0_ = 8.854 × 10^−3^ pF/mm and the air relative permittivity is *ε_r_*_2_ = 1.00053.

Obviously, the main task of a numerical design and calibration is to determine the specific values of these geometric and physical parameters, in which the radius *a*, initial parallel gap *g*, and original length *L* determine the spatial dimensions of the sensor to be designed and thus are determined by the specific use conditions of the sensor. As an example, it is assumed that *a* = 70 mm, *g* = 5 mm, and *L* = 40 mm, in which the membrane deflection due to its self-weight is assumed to be less than or equal to 5 mm. It should be noted that if the sensor is placed vertically (rather than horizontally as in Figure 1), then the initial parallel gap *g* should take zero; i.e., the most general case of use is considered here. In addition, the wind pressure *q*, that is, the transverse uniform loads *q* used in the derivation in Appendix A, is also determined by the specific use conditions of the sensor to be designed, and the wind pressure *q* to be measured is assumed here to be less than about 15,000 Pa.

The remaining geometric and physical parameters are parameters that need to be determined by trial and error, that is, first specifying the values of these parameters, then performing extensive numerical calculations with the closed-form solution derived in Appendix A, and then using the results of these numerical calculations to generate a scatter plot whose vertical coordinate is the wind pressure *q* and horizontal coordinate is the total capacitance *C* of the sensor. The reason why the wind pressure *q* is specified as the vertical coordinate variable and the capacitance *C* is specified as the horizontal coordinate variable is to meet the measurement principle of the wind pressure measurement system addressed here; that is, the wind pressure *q* applied to the parallel plate variable capacitor-based circular wind pressure sensor can be detected by measuring the capacitance *C* of the sensor subjected to wind pressure *q*. Therefore, the wind pressure measurement system requires an input/output analytical relationship that uses the capacitance *C* as an input variable and the wind pressure *q* as an output variable (called the capacitance–pressure (*C*–*p*) analytical relationship for short), rather than the pressure–capacitance (*p*–*C*) analytical relationship that uses the wind pressure *q* as an input variable and the capacitance *C* as an output variable (see Equation (4)).

Assuming that a circular membrane with a radius *a* = 70 mm, thickness *h* = 0.3 mm, Poisson’s ratio *v* = 0.45, Young’s modulus of elasticity *E* = 3.01 MPa, and yield strength *σ_y_* = 2 MPa is tentatively used, and the remaining other geometric and physical parameters are tentatively determined as *k* = 0.5 N/mm, Δ*l* = 5 mm (i.e., the self-weight of the movable electrode plate is equal to *k*Δ*l* = 2.5 N ≈ 0.25 kg), *t* = 0.1 mm, and *ε_r_*_1_ = 2.5 (the insulator layer is assumed to use polyethylene). It can be seen from Figure 2 that when the wind pressure *q* takes 163 Pa, the maximum deflection *w_m_* of the circular membrane is close to 10 mm, that is, the circular membrane is very close to the spring-reset movable electrode plate (due to *g* + Δ*l* = 10 mm). Therefore, based on the closed-form solution derived in Appendix A as well as the *p*–*C* analytical relationship given by Equation (4), the numerical calculations of the membrane/plate elastic contact problem dealt with in Appendix A may start from *q* = 164 Pa.

It can be found from Equation (A28) that *Q* = 0.012713, *K* = 0.176251, $\bar{L}$ = 0.071429, and *G* = 0.071429, where *q* = 164 Pa = 1.64 × 10^−4^ MPa (N/mm^2^), *a* = 70 mm, *E* = 3.01 MPa, *h* = 0.3 mm, *k* = 0.5 N/mm, Δ*l* = 5 mm, and *g* = 5 mm. Therefore, by simultaneously solving Equations (A51), (A52), and (A55), it can be found that the undetermined constants *c*_0_, *c*_1_, and *β* (*β* = (1 + *α*)/2 and *α* = *b*/*a*) are *c*_0_ = 0.023938, *c*_1_ = −0.004382, and *β* = 0.508089 (*α* = 0.016178 and *b* = 1.13246 mm), where *Q* = 0.012713, *K* = 0.176251, $\bar{L}$ = 0.071429, *G* = 0.071429, and *v* = 0.45. Further, with *c*_0_ = 0.023938, *c*_1_ = −0.004382, and *β* = 0.508089, it can be found from Equation (A49) that the dependent undetermined constant *d*_0_ is *d*_0_ = 0.109411. In addition, it can be found from Equation (A59) that the maximum membrane stress *σ_m_* is *σ_m_* = 0.075231 MPa, it can be found from Equation (A58) that the maximum membrane deflection *w_m_* is *w_m_* = 10.00132 mm, and finally it can be found from Equation (4) that the total capacitance *C* of the sensor is *C* = 3.8923, where *L* = 40 mm, *g* = 5 mm, *t* = 0.1 mm, *w_m_* = 10.00132 mm, *ε_r_*_1_ = 2.5, *ε_r_*_2_ = 1.00053, and *ε*_0_ = 8.854 × 10^−3^ pF/mm.

After finishing the calculations of the first step for *q* = 164 Pa, the value of the wind pressure *q* needs to be increased by an as-small-as-possible increment Δ*q* to continue the numerical calculations for *q* + Δ*q*. If the maximum membrane deflection *w_m_* calculated in this step is less than *L* (40 mm) and the maximum membrane stress *σ_m_* calculated in this step is less than *σ_y_* (2 MPa), then the value of the wind pressure *q* needs to be further increased by another increment Δ*q* to continue the numerical calculations for *q* + 2Δ*q*, and so on, until the maximum membrane deflection *w_m_* calculated is close to *L* (40 mm), or the maximum membrane stress *σ_m_* calculated is close to *σ_y_* (2 MPa). At this time, if the value of the wind pressure *q* is not yet close to 15,000 Pa, then the value of the membrane thickness *h* needs to be increased by an as-small-as-possible increment Δ*h* to repeat the above process of numerical calculations with *h* + Δ*h*. The condition that such a repeat can be stopped is that the maximum calculated membrane deflection *w_m_* is close to *L* (40 mm) and the value of the wind pressure *q* is close to 15,000 Pa but the maximum membrane stress *σ_m_* calculated is not yet close to *σ_y_* (2 MPa). The numerical calculations can be stopped, if this stop condition is met. The results of numerical calculations for this numerical example are listed in Table A1 in Appendix D.

**Figure 4:**
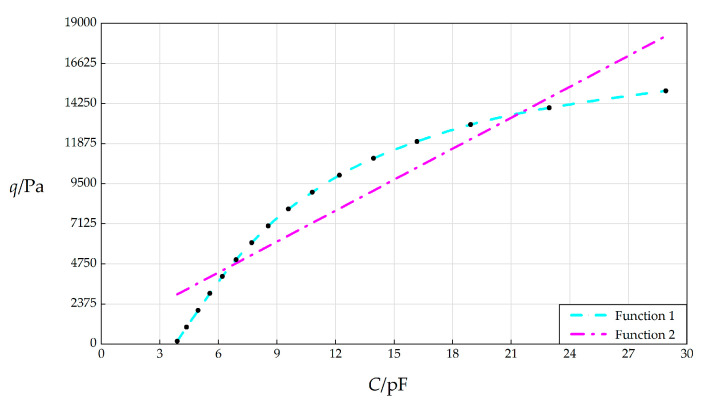
The variation in the applied pressure *q* with the total capacitances *C* when *a* = 70 mm, *h* = 0.3 mm, *t* = 0.1 mm, *E* = 3.01 MPa, *ν* = 0.45, *g* = 5 mm, Δ*l* = 5 mm, and *L* = 40 mm.

A scatter plot whose vertical coordinate is the wind pressure *q* and horizontal coordinate is the total capacitance *C* is shown in Figure 4, where the solid points represent the data pairs of pressure *q* and capacitance *C* in Table A1. “Function 1” and “Function 2”, i.e., the dashed curve and dot–dashed line in Figure 4, are the nonlinear and linear capacitance–pressure (*C*–*p*) analytical relationships, which are obtained based on least-square fitting for the numerical calculation values of the pressure *q* and capacitance *C* in Table A1. The fitted nonlinear and linear analytical expressions for “Function 1” and “Function 2” are listed in Table 1. Obviously, the wind pressure measurement system to be designed can adopt either a nonlinear or linear analytical expression as its *C*–*q* analytical relationship, where the linear one is simple and is therefore suitable for either analog or digital techniques, while the complex nonlinear analytical expression is only suitable for digital techniques.

It can be seen from Table A1 that the variation range of the input capacitance *C* is about 3.8923 pF~28.9153 pF, and the variation range of the output wind pressure *q* is about 164 Pa~15000 Pa. Therefore, a capacitance of 1fF can identify a wind pressure of about 0.593 Pa, that is, this wind pressure measurement system can use 1fF capacitance to recognize 0.593 Pa wind pressure.

However, if this calibration result of 0.593 Pa/fF does not meet the use requirements or technical needs, then the capacitance–pressure (*C*–*p*) analytical relationship of the measurement system needs to be regenerated by adjusting one or some of the design parameters, including the spring stiffness coefficient *k*, radius *a*, thickness *h* and *t*, Poisson’s ratio *v*, Young’s modulus of elasticity *E*, initial parallel gap *g*, and the initial spring compression Δ*l*, until the use requirements or technical needs are met. So, from this point of view, it is necessary to know in advance how changes in the design parameters will qualitatively and quantitatively affect the *C*–*q* analytical relationships, which will be addressed in the next section.

### 3.3. Effects of Changing Design Parameters on C–q Analytical Relationships

In this section, the values of the design parameters used in Section 3.2, i.e., *k* = 0.5 N/mm, *a* = 70 mm, *h* = 0.3 mm, *t* = 0.1 mm, *E* = 3.01 MPa, *ν* = 0.45, *g* = 5 mm, and Δ*l* = 5 mm, will be used as a benchmark for parameter adjustments here. The remaining parameters, which will be kept unchanged, are *L* = 40 mm, *ε_r_*_1_ = 2.5, *ε_r_*_2_ = 1.00053, and *ε*_0_ = 8.854×10^−3^ pF/mm. On the basis of this benchmark, the spring stiffness coefficient *k*, radius *a*, thickness *h* and *t*, Young’s modulus of elasticity *E*, Poisson’s ratio *v*, initial parallel gap *g*, and initial spring compression Δ*l* will be changed one by one to qualitatively and quantitatively investigate their effects on the capacitance–pressure (*C*–*p*) analytical relationships.

#### 3.3.1. Effect of Changing Spring Stiffness Coefficient *k* on *C*–*q* Relationships

**Figure 5:**
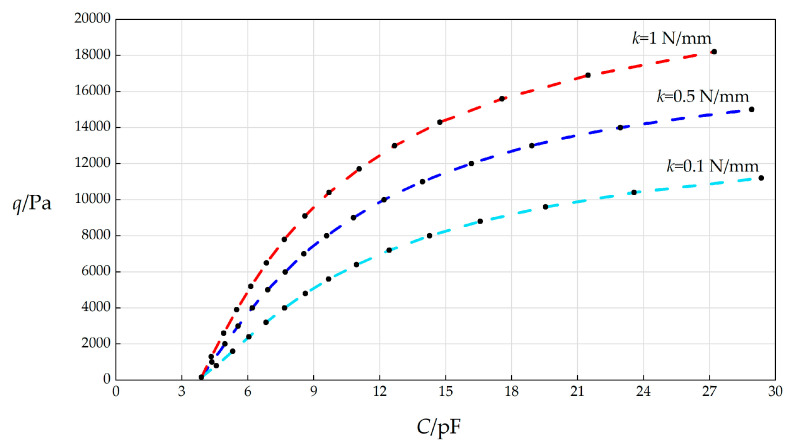
The effect of changing the spring stiffness coefficient *k* on the *C*–*q* relationships when *a =* 70 mm, *h* = 0.3 mm, *t* = 0.1 mm, *E* = 3.01 MPa, *ν* = 0.45, Δ*l* = 5 mm, *L* = 40 mm, and *k* takes 0.1 N/mm, 0.5 N/mm, and 1 N/mm, respectively.

In this section, the spring stiffness coefficient *k* will, respectively, take 0.1 N/mm, 0.5 N/mm, and 1 N/mm, and the other parameters remain unchanged, while the wind pressure *q* takes different values. The numerical calculation results are listed in Table A1 for *k* = 0.5 N/mm, in Table A2 for *k* = 0.1 N/mm, and in Table A3 for *k* = 1 N/mm. The *C*–*p* analytical relationships generated by the data in Table A1, Table A2 and Table A3 are shown in Figure 5.

From Figure 5, the qualitative effect of changing the spring stiffness coefficient *k* on the *C*–*p* analytical relationships can be concluded as follows: with the increase in *k*, the variation range of *q* increases, but the variation range of *C* decreases. The quantitative effect of changing *k* on *C*–*p* analytical relationships are analyzed as follows. The relative increase in *k* is (1 N/mm − 0.1 N/mm)/0.1 N/mm = 900%. It can be found from Table A2 and Table A3 that the relative increase in the variation range of *q* is [(18200 Pa − 164 Pa) − (11200 Pa − 164 Pa)]/(11200 Pa − 164 Pa) = 63.43%, and the relative increase in *C* is [(27.2152 pF − 3.8922 pF) − (29.3503 pF − 3.8927 pF)]/(29.3503 pF − 3.8927 pF) = −8.38%. Therefore, the degree of influence of increasing *k* on the variation range of *q* is 63.43%/900% = 7.05%, and the degree of influence of increasing *k* on the variation range of *C* is −8.38%/900% = −0.93%. Therefore, it can be concluded that a 1% increase in the spring stiffness coefficient *k* will result in a 7.05% increase in the variation range of the output wind pressure *q* and a 0.93% decrease in the variation range of the input capacitance *C*.

#### 3.3.2. Effect of Changing Outer Radius *a* on *C*–*q* Relationships

**Figure 6:**
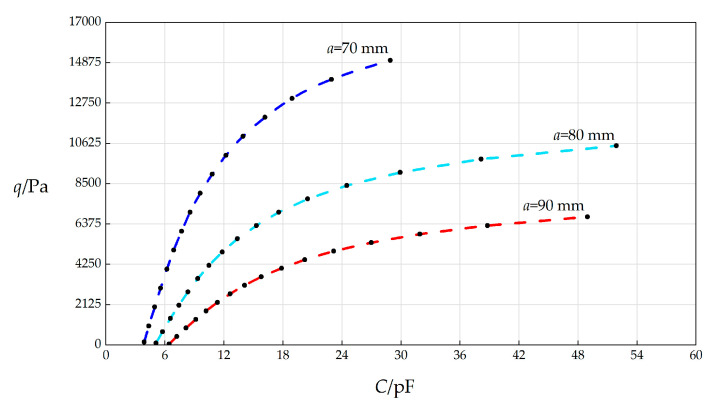
The effect of changing the circular membrane radius *a* on *C*–*q* relationships when *k =* 0.5 N/mm, *h* = 0.3 mm, *t* = 0.1 mm, *E* = 3.01 MPa, *ν* = 0.45, Δ*l* = 5 mm, *L* = 40 mm, and *a* takes 70 mm, 80 mm, and 90 mm, respectively.

In this section, the radius *a* will, respectively, take 70 mm, 80 mm, and 90 mm, and the other parameters remain unchanged, while the wind pressure *q* takes different values. The numerical calculation results are listed in Table A1 for *a* = 70 mm, in Table A4 for *a* = 80 mm, and in Table A5 for *a* = 90 mm. The *C*–*p* analytical relationships generated by the data in Table A1, Table A4 and Table A5 are shown in Figure 6.

From Figure 6, the qualitative effect of changing *a* on *C*–*p* analytical relationships can be concluded as follows: with the increase in *a*, the variation range of *q* decreases, but the variation range of *C* increases. The quantitative effect of changing *a* on *C*–*p* analytical relationships is analyzed as follows. The relative increase in *a* is (90 mm − 70 mm)/70 mm = 28.57%. It can be found from Table A1 and Table A5 that the relative increase in the variation range of *q* is [(6750 Pa − 60 Pa) − (15000 Pa − 164 Pa)]/(15000 Pa − 164 Pa) = −54.91%, and the relative increase in *C* is [(48.9625 pF − 6.4340 pF) − (28.9153 pF − 3.8923 pF)]/(28.9153 pF − 3.8923 pF) = 69.96%. Therefore, the influence degree of increasing *a* on the variation range of *q* is −54.91%/28.57% = −192.19%, and the influence degree of increasing *a* on the variation range of *C* is 69.96%/28.57% = 244.87%. Therefore, it can be concluded that a 1% increase in the radius *a* will result in a 192.19% decrease in the variation range of the output wind pressure *q* and a 244.87% increase in the variation range of the input capacitance *C*.

#### 3.3.3. Effect of Changing Membrane Thickness *h* on *C*–*q* Relationships

**Figure 7:**
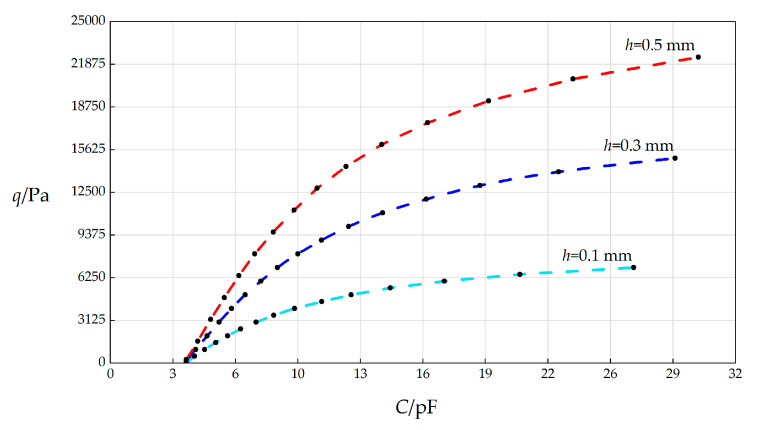
The effect of changing the circular membrane thickness *h* on the *C*–*q* relationships when *k =* 0.5 N/mm, *a* = 70 mm, *t* = 0.1 mm, *E* = 3.01 MPa, *ν* = 0.45, Δ*l* = 5 mm, *L* = 40 mm, and *h* takes 0.1 mm, 0.3 mm, and 0.5 mm, respectively.

In this section, the membrane thickness *h* will, respectively, take 0.1 mm, 0.3 mm, and 0.5 mm, and the other parameters remain unchanged, while the wind pressure *q* takes different values. The numerical calculation results are listed in Table A1 for *h* = 0.3 mm, in Table A6 for *h* = 0.1 mm, and in Table A7 for *h* = 0.5 mm. The *C*–*p* analytical relationships generated by the data in Table A1, Table A6 and Table A7 are shown in Figure 7.

From Figure 7, the qualitative effect of changing *h* on *C*–*p* analytical relationships can be concluded as follows: with the increase in *h*, the variation range of *q* increases and the variation range of *C* slightly increases. The quantitative effect of changing *h* on *C*–*p* analytical relationships are analyzed as follows. The relative increase in *h* is (0.5 mm − 0.1 mm)/0.1 mm = 400%. It can be found from Table A6 and Table A7 that the relative increase in the variation range of *q* is [(22400 Pa − 272 Pa) − (7000 Pa − 54.5 Pa)]/(7000 Pa − 54.5 Pa) = 218.59%, and the relative increase in *C* is [(30.0945 pF − 3.8921 pF) − (26.7918 pF − 3.8922 pF)]/(26.7918 pF − 3.8922 pF) = 14.42%. Therefore, the influence degree of increasing *h* on the variation range of *q* is 218.59%/400% = 54.65%, and the influence degree of increasing *h* on the variation range of *C* is 14.42%/400% = 3.61%. Therefore, it can be concluded that a 1% increase in the membrane thickness *h* will result in a 54.65% increase in the variation range of the output wind pressure *q* and a 3.61% increase in the variation range of the input capacitance *C*.

#### 3.3.4. Effect of Changing Insulator Layer Thickness *t* on *C*–*p* Relationships

**Figure 8:**
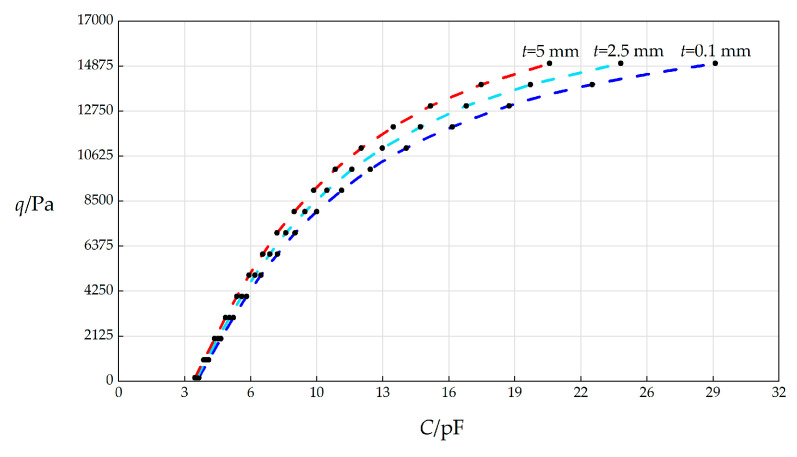
The effect of changing the insulator layer thickness *t* on the *C*–*q* relationships when *k =* 0.5 N/mm, *a* = 70 mm, *h* = 0.3 mm, *E* = 3.01 MPa, *ν =* 0.45, *g* = 5 mm, Δ*l* = 5 mm, *L* = 40 mm, and *t* takes 0.1 mm, 2.5 mm, and 5 mm, respectively.

In this section, the insulator layer thickness *t* will, respectively, take 0.1 mm, 2.5 mm, and 5 mm, and the other parameters remain unchanged, while the wind pressure *q* takes different values. The numerical calculation results are listed in Table A1 for *t* = 0.1 mm, in Table A8 for *t* = 2.5 mm, and in Table A9 for *t* = 5 mm. The *C*–*p* analytical relationships generated by the data in Table A1, Table A8, and Table A9 are shown in Figure 8.

From Figure 8, the qualitative effect of changing *t* on *C*–*p* analytical relationships can be concluded as follows: with the increase in *t*, the variation range of *q* does not change but the variation range of *C* decreases. The quantitative effect of changing *t* on *C*–*p* analytical relationships are analyzed as follows. The relative increase in *t* is (5 mm − 0.1 mm)/0.1 mm = 4900%. It can be found from Table A1 and Table A9 that the relative increase in the variation range of *q* is [(15000 Pa − 164 Pa) − (15000 Pa − 164 Pa)]/(15000 Pa − 164 Pa) = 0, and the relative increase in *C* is [(20.8773 pF − 3.7005 pF) − (28.9153 pF − 3.8923 pF)]/(28.9153 pF − 3.8923 pF) = −31.36%. Therefore, the influence degree of increasing *t* on the variation range of *q* is equal to zero, and the influence degree of increasing *t* on the variation range of *C* is −31.36%/4900% = −0.64%. Therefore, it can be concluded that a 1% increase in the insulator layer thickness *t* will result only in a 0.64% decrease in the variation range of the input capacitance *C*.

#### 3.3.5. Effect of Changing Young’s Modulus of Elasticity *E* on *C*–*q* Relationships

**Figure 9:**
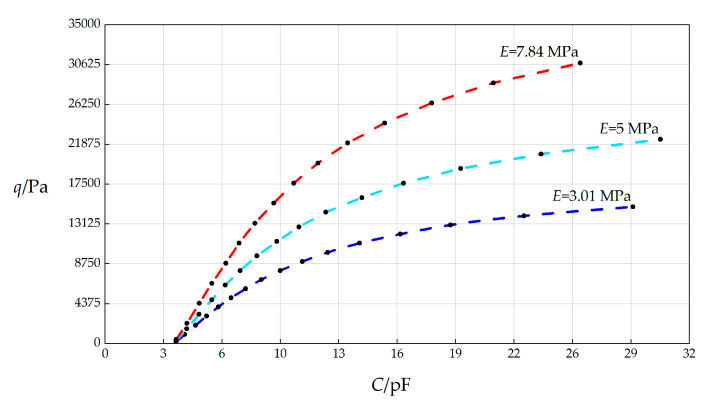
The effect of changing Young’s modulus of elasticity *E* on the *C*–*q* relationships when *k =* 0.5 N/mm, *a* = 70 mm, *h* = 0.3 mm, *t* = 0.1 mm, *ν* = 0.45, Δ*l* = 5 mm, *L* = 40 mm, and *E* takes 3.01 MPa, 5 MPa, and 7.84 MPa, respectively.

In this section, the spring stiffness coefficient *k* will, respectively, take 3.01 MPa, 5 MPa, and 7.84 MPa, and the other parameters remain unchanged, while the wind pressure *q* takes different values. The numerical calculation results are listed in Table A1 for *E* = 3.01 MPa, in Table A10 for *E* = 5 MPa, and in Table A11 for *E* = 7.84 MPa. The *C*–*p* analytical relationships generated by the data in Table A1, Table A10, and Table A11 are shown in Figure 9.

From Figure 9, the qualitative effect of changing *E* on *C*–*p* analytical relationships can be concluded as follows: with the increase in *E*, the variation range of *q* increases, but the variation range of *C* decreases. The quantitative effect of changing *E* on *C*–*p* analytical relationships are analyzed as follows. The relative increase in *E* is (7.84 MPa − 3.01 MPa)/3.01 MPa = 160.47%. It can be found from Table A1 and Table A11 that the relative increase in the variation range of *q* is [(30800 Pa − 425.5 Pa) − (15000 Pa − 164 Pa)]/(15000 Pa − 164 Pa) = 104.74%, and the relative increase in *C* is [(26.0298 pF − 3.8922 pF) − (28.9153 pF − 3.8923 pF)]/(28.9153 pF − 3.8923 pF) = −11.53%. Therefore, the influence degree of increasing *E* on the variation range of *q* is 104.74%/160.47% = 65.27%, and the influence degree of increasing *E* on the variation range of *C* is −8.38%/160.47% = −7.19%. Therefore, it can be concluded that a 1% increase in the Young’s modulus of elasticity *E* will result in a 65.27% increase in the variation range of the output wind pressure *q* and a 7.19% decrease in the variation range of the input capacitance *C*.

#### 3.3.6. Effect of Changing Poisson’s Ratio *v* on *C*–*q* Relationships

**Figure 10:**
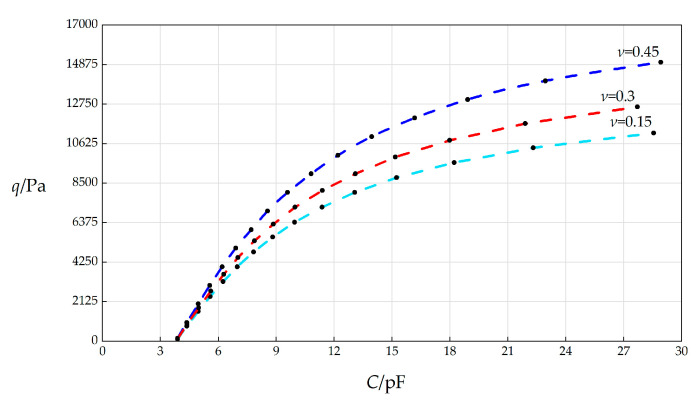
The effect of changing Poisson’s ratio *ν* on the *C*–*q* relationships when *k =* 0.5 N/mm, *a* = 70 mm, *h* = 0.3 mm, *t* = 0.1 mm, *E* = 3.01 MPa, Δ*l* = 5 mm, *L* = 40 mm, and *ν* takes 0.15, 0.3, and 0.45, respectively.

In this section, the Poisson’s ratio *v* will, respectively, take 0.15, 0.3, and 0.45, and the other parameters remain unchanged, while the wind pressure *q* takes different values. The numerical calculation results are listed in Table A1 for *v* = 0.45, in Table A12 for *v* = 0.15, and in Table A13 for *v* = 0.3. The *C*–*p* analytical relationships generated by the data in Table A1, Table A12, and Table A13 are shown in Figure 10.

From Figure 10, the qualitative effect of changing *v* on *C*–*p* analytical relationships can be concluded as follows: with the increase in *v*, the variation range of *q* increases, but the variation range of *C* hardly changes. The quantitative effect of changing *v* on *C*–*p* analytical relationships are analyzed as follows. The relative increase in *v* is (0.45 − 0.15)/0.15 = 200%. It can be found from Table A1 and Table A12 that the relative increase in the variation range of *q* is [(15000 Pa − 164 Pa) − (11200 Pa − 114 Pa)]/(11200 Pa − 164 Pa) = 33.83%, and the relative increase in *C* is [(28.9153 pF − 3.8923 pF) − (28.5514 pF − 3.8921 pF)]/(28.5514 pF − 3.8921 pF) = 1.47%. Therefore, the influence degree of increasing *v* on the variation range of *q* is 33.83%/200% = 16.91%, and the influence degree of increasing *v* on the variation range of *C* is 1.47%/200% = 0.74%. Therefore, it can be concluded that a 1% increase in the Poisson’s ratio *v* will result in a 16.91% increase in the variation range of the output wind pressure *q* and a 0.74% decrease in the variation range of the input capacitance *C*.

#### 3.3.7. Effect of Changing Initially Parallel Gap *g* on *C*–*q* Relationships

**Figure 11:**
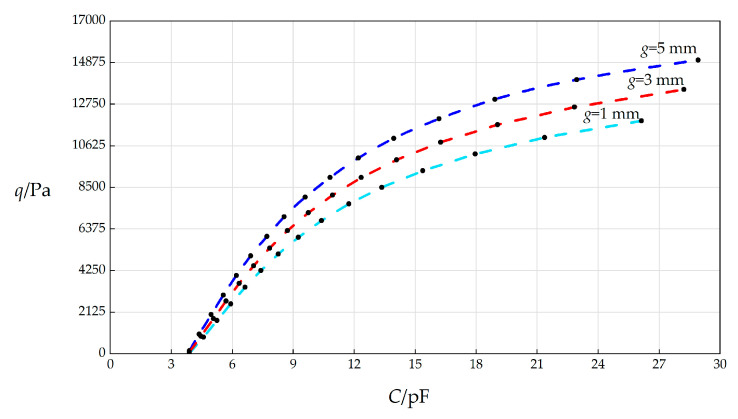
The effect of changing the initially parallel gap *g* on the *C*–*q* relationships when *k =* 0.5 N/mm, *a* = 70 mm, *h* = 0.3 mm, *t* = 0.1 mm, *E* = 3.01 MPa, *ν =* 0.45, Δ*l* = 5 mm, *L* = 40 mm, and *g* takes 1 mm, 3 mm, and 5 mm, respectively.

In this section, the initially parallel gap *g* will, respectively, take 1 mm, 3 mm, and 5 mm, and the other parameters remain unchanged, while the wind pressure *q* takes different values. The numerical calculation results are listed in Table A1 for *g* = 5 mm, in Table A14 for *g* = 1 mm, and in Table A15 for *g* = 3 mm. The *C*–*p* analytical relationships generated by the data in Table A1, Table A14, and Table A15 are shown in Figure 11.

From Figure 11, the qualitative effect of changing *g* on *C*–*p* analytical relationships can be concluded as follows: with the increase in *g*, the variation ranges of both *q* and *C* slightly increase. The quantitative effect of changing *g* on *C*–*p* analytical relationships are analyzed as follows. The relative increase in *g* is (5 mm − 1 mm)/1 mm = 400%. It can be found from Table A1 and Table A14 that the relative increase in the variation range of *q* is [(15000 Pa − 164 Pa) − (11900 Pa − 35.6 Pa)]/(11900 Pa − 35.6 Pa) = 25.05%, and the relative increase in *C* is [(28.9153 pF − 3.8923 pF) − (26.13 pF − 3.8922 pF)]/(26.13 pF − 3.8922 pF) = 12.52%. Therefore, the influence degree of increasing *g* on the variation range of *q* is 25.05%/400% = 6.26%, and the influence degree of increasing *g* on the variation range of *C* is 12.52%/400% = 3.13%. Therefore, it can be concluded that a 1% increase in the initially parallel gap *g* will result in a 6.26% increase in the variation range of the output wind pressure *q* and a 3.13% increase in the variation range of the input capacitance *C*.

#### 3.3.8. Effect of Changing Initial Spring Compression Δ*l* on *C*–*q* Relationships

**Figure 12:**
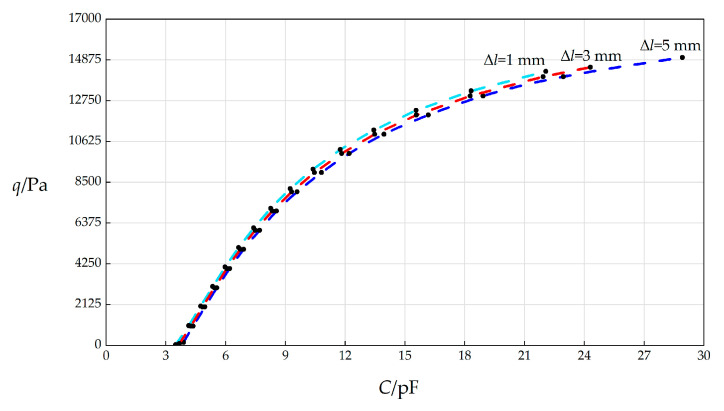
The effect of changing the initial compressed length Δ*l* of the spring on the *C*–*q* relationships when *k =* 0.5 N/mm, *a* = 70 mm, *h* = 0.3 mm, *t* = 0.1 mm, *E* = 3.01 MPa, *ν =* 0.45, *g* = 5 mm, *L* = 40 mm, and Δ*l* takes 1 mm, 3 mm, and 5 mm, respectively.

In this section, the initial spring compression Δ*l* will, respectively, take 1 mm, 3 mm, and 5 mm, and the other parameters remain unchanged, while the wind pressure *q* takes different values. The numerical calculation results are listed in Table A1 for Δ*l* = 5 mm, in Table A16 for Δ*l* = 1 mm, and in Table A17 for Δ*l* = 3 mm. The *C*–*p* analytical relationships generated by the data in Table A1, Table A16, and Table A17 are shown in Figure 12.

From Figure 12, the qualitative effect of changing Δ*l* on *C*–*p* analytical relationships can be concluded as follows: with the increase in Δ*l*, the variation range of *q* hardly changes, while the variation range of *C* slightly increases. The quantitative effect of changing Δ*l* on *C*–*p* analytical relationships are analyzed as follows. The relative increase in Δ*l* is (5 mm − 1 mm)/1 mm = 400%. It can be found from Table A1 and Table A16 that the relative increase in the variation range of *q* is [(15000 Pa − 164 Pa) − (15300 Pa − 35.5 Pa)]/(15300 Pa − 35.5 Pa) = −2.81%, and the relative increase in *C* is [(28.9153 pF − 3.8923 pF) − (27.484 pF − 3.4933 pF)]/(27.484 pF − 3.4933 pF) = 4.30%. Therefore, the influence degree of increasing Δ*l* on the variation range of *q* is −2.81%/400% = −0.70%, and the influence degree of increasing Δ*l* on the variation range of *C* is −4.30%/400% = 1.08%. Therefore, it can be concluded that a 1% increase in the initial spring compression Δ*l* will result in a 0.70% decrease in the variation range of the output wind pressure *q* and a 1.08% increase in the variation range of the input capacitance *C*.

## 4. Concluding Remarks

In this paper, a theoretical study on a parallel plate variable capacitor-based circular wind pressure sensor is presented, where the key theories (the closed-form solution and input/output analytical relationship) necessary to design such wind pressure sensors are given, the validity of the closed-form solution is proved, and a numerical example is given to demonstrate how to use the closed-form solution and input/output analytical relationship for numerical design and calibration. In addition, the qualitative and quantitative effects of changing design parameters on capacitance–pressure (*C*–*q*) analytical relationships are comprehensively investigated. To facilitate the design and development of such wind pressure sensors, the quantitative effects of changing design parameters on *C*–*q* analytical relationships are summarized as follows.

♦ A 1% increase in the spring stiffness coefficient *k* will give rise to a 7.05% increase in the variation range of the output wind pressure *q* and a 0.93% decrease in the variation range of the input capacitance *C*.
♦ A 1% increase in the radius *a* will give rise to a 192.19% decrease in the variation range of the output wind pressure *q* and a 244.87% increase in the variation range of the input capacitance *C*.
♦ A 1% increase in the membrane thickness *h* will give rise to a 54.65% increase in the variation range of the output wind pressure *q* and a 3.61% increase in the variation range of the input capacitance *C*.
♦ A 1% increase in the insulator layer thickness *t* will give rise to only a 0.64% decrease in the variation range of the input capacitance *C*.
♦ A 1% increase in the Young’s modulus of elasticity *E* will give rise to a 65.27% increase in the variation range of the output wind pressure *q* and a 7.19% decrease in the variation range of the input capacitance *C*.
♦ A 1% increase in the Poisson’s ratio *v* will give rise to a 16.91% increase in the variation range of the output wind pressure *q* and a 0.74% decrease in the variation range of the input capacitance *C*.
♦ A 1% increase in the initially parallel gap *g* will give rise to a 6.26% increase in the variation range of the output wind pressure *q* and a 3.13% increase in the variation range of the input capacitance *C*.
♦ A 1% increase in the initial spring compression Δ*l* will give rise to a 0.70% decrease in the variation range of the output wind pressure *q* and a 1.08% increase in the variation range of the input capacitance *C*.

According to the above quantitative effects of changing design parameters on capacitance–pressure (*C*–*q*) analytical relationships, if the *C*–*q* analytical relationship to be calibrated does not meet the use or technical requirements, the design parameter that has a greater effect on *C*–*q* analytical relationships should be first adjusted to increase or decrease the variation range of the output wind pressure *q* or the input capacitance *C*.

The work presented here is mainly devoted to providing the basic or general theory needed for the numerical design and calibration of such wind pressure sensors. So, further research is still needed, especially experimental studies combined with the development of well-defined sensors. The experimental tests of the wind pressure sensor to be developed should be carried out in a wind tunnel whose cross-section is at least three times larger than the area of the sensor, such that the sensor is similar to operating in a natural wind environment.

## Figures and Tables

**Table 1:** The analytical expressions of “Function 1” and “Function 2” in Figure 4 and the variation ranges of the output pressure *q* and input capacitance *C*.

Functions	*C*/pF	*q*/Pa	Analytical Expressions
Function 1	3.8923~28.9153	164~15000	*q* = −0.0004652*C*^5^ − 0.01513 *C*^4^ + 3.677 *C*^3^ − 156.7 *C*^2^ + 2991*C* − 9363
Function 2	3.8923~28.9153	164~15000	*q* = 611.5*C* + 569.2

**Figure A2:**
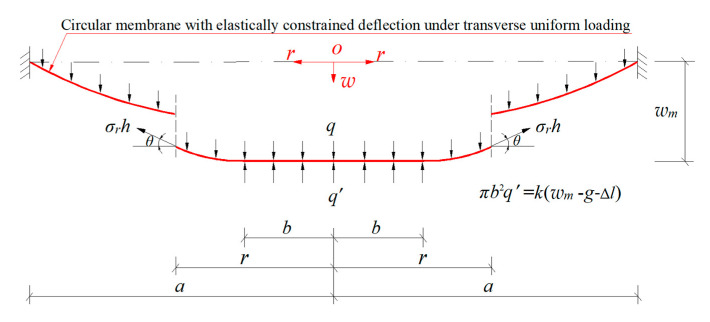
Sketch of the free body with radius *r* (*b* ≤ *r* ≤ *a*).

## Data Availability

The original contributions presented in this study are included in the article. Further inquiries can be directed to the corresponding author(s).

## Appendix A. Closed-Form Solution of Membrane/Plate Elastic Contact Problem

Suppose that the Young’s modulus of elasticity, Poisson’s ratio, thickness, and radius of the initially flat and peripherally fixed circular membrane used in Figure 1 are denoted by *E*, *v*, *h*, and *a*, respectively, and the initial parallel gap *g* between the initially flat and peripherally fixed circular membrane and the spring-reset movable electrode plate is greater than or equal to the deflection due to the self-weight of the circular membrane.

The wind-driven circular membrane freely and elastically behaves before it touches the spring-reset movable electrode plate, as shown in Figure A1a, where *o*, *r*, *φ*, and *w* denote the coordinate origin, radial, circumferential, and transverse coordinates of the introduced cylindrical coordinate system (*r*, *φ*, *w*), respectively; the coordinate origin ***o*** is at the centroid of the geometric middle plane (the dash–dotted line) of the circular membrane that is initially flat and peripherally fixed, the polar plane (*r*, *φ*) is located in the plane in which the geometric middle plane is located; and *w_m_* denotes the maximum deflection of the circular membrane under the transverse uniform loading of the wind pressure *q*. In addition, *w* also denotes the membrane deflection or transverse displacement of the circular membrane under the transverse uniform loading of the wind pressure *q*, and the circumferential coordinate *φ* cannot be shown due to the profile. The large deflection elastic behavior of the circular membrane with free deflection under the transverse uniform loading of the wind pressure *q* (see Figure A1a) has been well formulated mathematically, and its closed-form solution can be found in [55].

As the wind pressure *q* intensifies, the wind-driven large deflection circular membrane will be in contact elastically with the spring-reset movable electrode plate (its deflection is elastically constrained by the spring-reset movable electrode plate), as shown in Figure A1b, where the contact radius between the spring-reset movable electrode plate and the wind-driven large deflection circular membrane is denoted by *b*. When the interaction between the wind-driven large deflection circular membrane and the spring-reset movable electrode plate reaches static equilibrium, the spring is compressed by Δ*L* (Δ*L* = *w_m_* − *g*, see Figure A1b) from its original length *L*. Such a membrane/plate interaction can be analyzed by introducing a membrane/plate interaction force *q*’ that is uniformly distributed over the contact area π*b*^2^, as shown in Figure A1c, where the upward force perpendicular to the polar plane (*r*, *φ*) acting on the movable electrode plate is only the restoring force *F* (*F* = *k*Δ*L* = *k*(*w_m_* − *g*)) of the compressed spring, and the downward forces are the external force π*b*^2^ *q*’ and the self-weight *k*Δ*l* of the movable electrode plate. So, the static equilibrium condition of the movable electrode plate under the joint actions of π*b*^2^ *q*’, *k*Δ*l* and *F* is π*b*^2^ *q*’ + *k*Δ*l* = *F* = *k*Δ*L* = *k*(*w_m_* − *g*), as seen in Figure A1c. This equilibrium condition will be used for analytically solving the large deflection problem of membrane/plate elastic contact in Figure A1b or Figure A1c.

A free body with a radius *r* (*b* ≤ *r* ≤ *a*) is taken from the central portion of the wind-driven large deflection circular membrane that is being in contact with the spring-reset movable electrode plate, as shown in Figure A2, where *σ_r_* represents the radial stress at *r*, *σ_r_h* is the membrane force that is acting on the boundary of the radius *r*, and *θ* denotes the meridional rotation angle of the deflected circular membrane at *r*. In the vertical direction perpendicular to the polar plane (*r*, *φ*), the static equilibrium condition of this free body under the transverse uniform loading of the wind pressure *q* is

(A1) $$2\pi r\sigma_{r}h\sin\theta -\pi r^{2}q+\pi b^{2}q^{\prime }=0,$$

where 2*πrσ_r_h*sin*θ* is the vertical component of the radial force acting on the boundary of *r*. From the physical phenomenon of the contact problem in Figure A1, it can be found that *θ* = 0 at *r* = *b*, that is, sin*θ* = 0 at *r* = *b*. Therefore, after applying the condition of sin*θ* = 0 at *r* = *b* to Equation (A1), it can be easily found that *q*’ = *q*. So, Equation (A1) can be reduced to

(A2) $$2\pi r\sigma_{r}h\sin\theta -\pi (r^{2}-b^{2})q=0,$$

where

(A3) $$\sin\theta =1/\sqrt{1+1/\tan^{2}\theta }=1/\sqrt{1+1/{\left(-dw/dr\right)}^{2}}.$$

From Equations (A2) and (A3), the so-called out-of-plane equilibrium equation can be finally written as

(A4) $$2r\sigma_{r}h-(r^{2}-b^{2})q\sqrt{1+1/{\left(-dw/dr\right)}^{2}}=0$$

In the horizontal direction parallel to the polar plane (*r*, *φ*), there are only the circumferential stress *σ_t_* and the horizontal component of the radial stress *σ_r_*. The relationship between radial and circumferential stresses at any point on the circular membrane within the range of *b* ≤ *r* ≤ *a*, i.e., the in-plane equilibrium equation, is given by [55]

(A5) $$\frac{d}{dr}(r\sigma_{r})-\sigma_{t}=0.$$

For large deflection membranes, the so-called geometric equations, that is, the relationships between the radial and circumferential strains, *e_r_* and *e_t_*, and the radial and transverse displacements, *u* and *w*, may be written as [55]

(A6) $$e_{r}=\frac{du}{dr}+\frac{1}{2}{\left(-\frac{dw}{dr}\right)}^{2},$$

(A7) $$e_{t}=\frac{u}{r}.$$

Moreover, for the circular membrane of Hooke-type materials, the physical equations, i.e., the stress and strain relationships, follow Hooke’s law [55]:

(A8) $$\sigma_{r}=\frac{E}{1-\nu^{2}}(e_{r}+\nu e_{t}),$$

(A9) $$\sigma_{t}=\frac{E}{1-\nu^{2}}(e_{t}+\nu e_{r}).$$

From Equations (A6)–(A9), it is found that

(A10) $$\sigma_{r}=\frac{E}{1-\nu^{2}}[\frac{du}{dr}+\frac{1}{2}{\left(-\frac{dw}{dr}\right)}^{2}+\nu \frac{u}{r}],$$

(A11) $$\sigma_{t}=\frac{E}{1-\nu^{2}}[\frac{u}{r}+\nu \frac{du}{dr}+\frac{\nu }{2}{\left(-\frac{dw}{dr}\right)}^{2}].$$

By means of Equations (A10), (A11), and (A5), one has

(A12) $$\frac{u}{r}=\frac{1}{E}(\sigma_{t}-\nu \sigma_{r})=\frac{1}{E}[\frac{d}{dr}(r\sigma_{r})-\nu \sigma_{r}].$$

After substituting the *u* of Equation (A12) into Equation (A10), it is found that

(A13) $$r\frac{d}{dr}[\frac{1}{r}\frac{d}{dr}(r^{2}\sigma_{r})]+\frac{E}{2}{\left(-\frac{dw}{dr}\right)}^{2}=0.$$

Equations (A4) and (A13) are two equations for solving the radial stress *σ_r_* and transverse displacement *w* of the membrane/plate non-contact region of *b* ≤ *r* ≤ *a*. With the known solutions of *σ_r_* and *w*, the other solutions of *σ_t_*, *e_r_*, *e_t_*, and *u* can then be easily obtained from Equations (A5), (A6), (A7), and (A12). Therefore, the problem of the membrane/plate non-contact region of *b* ≤ *r* ≤ *a* can be solved analytically.

On the other hand, in the membrane/plate contact region of 0 ≤ *r* ≤ *b*, since d*w*/d*r* = 0, Equations (A6) and (A7) can be reduced to

(A14) $$e_{r}=\frac{du}{dr},$$

(A15) $$e_{t}=\frac{u}{r}.$$

After substituting d*w*/d*r* = 0 into Equations (A10) and (A11), it is found that

(A16) $$\sigma_{r}=\frac{E}{1-\nu^{2}}(\frac{du}{dr}+\nu \frac{u}{r}),$$

(A17) $$\sigma_{t}=\frac{E}{1-\nu^{2}}(\frac{u}{r}+\nu \frac{du}{dr}).$$

Substituting Equations (A16) and (A17) into Equation (A5) yields

(A18) $$r\frac{d^{2}u}{dr^{2}}+\frac{du}{dr}-\frac{u}{r}=0.$$

Since Equation (A18) satisfies the form of the Euler equation, its general solution may be written as

(A19) $$u(r)=C_{1}r+C_{2}\frac{1}{r},$$

where *C*_1_ and *C*_2_ are two undetermined constants. Obviously, the radial displacement at *r* = 0 is equal to zero, i.e., *u*(0) = 0. Since Equation (A19) must satisfy the condition of *u*(0) = 0, then *C*_2_ must be identically equal to zero, i.e., *C*_2_ ≡ 0. If the radial displacement at *r* = *b* is denoted by *u*(*b*), then it can be obtained from Equation (A19) that *C*_1_ = *u*(*b*)/*b*, due to *C*_2_ ≡ 0. This results in

(A20) $$u(r)=\frac{u(b)}{b}r.$$

If substituting Equation (A20) into Equations (A14)–(A17), then

(A21) $$e_{r}=e_{t}=\frac{u(b)}{b},$$

(A22) $$\sigma_{r}=\sigma_{t}=\frac{E}{1-\nu }\frac{u(b)}{b}.$$

It can be seen from Equations (A21) and (A22) that in the membrane/plate contact region of 0 ≤ *r* ≤ *b*, the strain is uniformly distributed and the radial strain is always equal to the circumferential strain. Also, the stress is uniformly distributed and the radial stress is always equal to the circumferential stress. Note, however, that the contact radius *b* and radial displacement *u*(*b*), like the radial stress *σ_r_* and transverse displacement *w* of the membrane/plate non-contact region of *b* ≤ *r* ≤ *a*, are still quantities to be determined at this point.

Obviously, the changes in stress, strain, and displacement in the membrane/plate contact region of 0 ≤ *r* ≤ *b* are continuous with those in the membrane/plate non-contact region of *b* ≤ *r* ≤ *a*. The boundary conditions and continuous conditions, which are used for determining the stress, strain, and displacement of the membrane/plate non-contact region of *b* ≤ *r* ≤ *a* and the contact radius *b* and radial displacement *u*(*b*) of the membrane/plate contact region of 0 ≤ *r* ≤ *b*, are as follows:

(A23) w=0 at r=a,

(A24) $$e_{t}=\frac{u}{r}=\frac{1}{E}[\frac{d}{dr}(r\sigma_{r})-\nu \sigma_{r}]=0\;\mathrm{at}\;r=a,$$

(A25) $${\left(e_{t}\right)}_{A}={\left(e_{t}\right)}_{B}=\frac{u(b)}{b}\;\mathrm{at}\;r=b,$$

(A26) $${\left(\sigma_{r}\right)}_{A}={\left(\sigma_{r}\right)}_{B}=\frac{E}{1-\nu }\frac{u(b)}{b}\;\mathrm{at}\;r=b,$$

where the subscripts *A* and *B* denote the membrane/plate non-contact and contact regions on the two sides of the inter-connecting circle of *r* = *b*. In addition, the equilibrium condition of the movable electrode plate under the joint actions of π*b*^2^ *q*’, *k*Δ*l*, and *F*, that is, π*b*^2^ *q*’ + *k*Δ*l* = *k*(*w_m_* − *g*) (see Figure A1c), must also be used. This equilibrium condition can be reduced to, from the derivation result *q*’ = *q* (see the derivation between Equations (A1) and (A2)),

(A27) $$\pi b^{2}q=k(w_{m}-g-\Delta l),$$

where the maximum membrane deflection *w_m_* is equal to the membrane deflection *w* at *r* = *b*, i.e., *w_m_* = *w*(*b*).

Now, let us introduce the following nondimensionalization:

(A28) $$Q=\frac{qa}{Eh},W=\frac{w}{a},S_{r}=\frac{\sigma_{r}}{E},S_{t}=\frac{\sigma_{t}}{E},x=\frac{r}{a},\alpha =\frac{b}{a},K=\frac{k}{\pi Eh},\bar{L}=\frac{\Delta l}{a},G=\frac{g}{a},$$

where *b* ≤ *r* ≤ *a*, i.e., *α* ≤ *x* ≤ 1. Further, use Equation (A28) to transform Equations (A4) and (A13) and Equations (A23)–(A27) into

(A29) $$4x^{2}S_{r}^{2}{\left(-\frac{dW}{dx}\right)}^{2}-{\left(x^{2}-\alpha^{2}\right)}^{2}Q^{2}[1+{\left(-\frac{dW}{dx}\right)}^{2}]=0,$$

(A30) $$x^{2}\frac{d^{2}S_{r}}{dx^{2}}+3x\frac{dS_{r}}{dx}+\frac{1}{2}{\left(-\frac{dW}{dx}\right)}^{2}=0,$$

(A31) W=0 at x=1,

(A32) $$S_{r}-\nu S_{r}+x\frac{dS_{r}}{dx}=0\;\mathrm{at}\;x=1,$$

(A33) $$S_{r}-\nu S_{r}+x\frac{dS_{r}}{dx}=\frac{u(b)}{b}\;\mathrm{at}\;x=\alpha ,$$

(A34) $$S_{r}=\frac{1}{1-\nu }\frac{u(b)}{b}\;\mathrm{at}\;x=\alpha ,$$

(A35) $$\alpha^{2}Q=K(W_{m}-G-\bar{L}),$$

where *W_m_* = *W*(*α*). Eliminating d*W*/d*x* from Equations (A29) and (A30) gives

(A36) $$[8x^{4}S_{r}^{2}-2x^{2}{\left(x^{2}-\alpha^{2}\right)}^{2}Q^{2}]\frac{d^{2}S_{r}}{dx^{2}}+[24x^{3}S_{r}^{2}-6x{\left(x^{2}-\alpha^{2}\right)}^{2}Q^{2}]\frac{dS_{r}}{dx}+{\left(x^{2}-\alpha^{2}\right)}^{2}Q^{2}=0.$$

In order to use the power series method for differential equations, *S_r_* and *W* have to be expanded into the power series of *x*-*β*, where *β* = (1 + *α*)/2, i.e.,

(A37) $$S_{r}=\sum_{i=0}^{\infty }c_{i}{\left(x-\beta \right)}^{i},$$

(A38) $$W=\sum_{i=0}^{\infty }d_{i}{\left(x-\beta \right)}^{i},$$

For convenience, we further introduce *X* = *x* − *β* and reduce Equations (A37) and (A38) to

(A39) $$S_{r}=\sum_{i=0}^{\infty }c_{i}X^{i},$$

(A40) $$W=\sum_{i=0}^{\infty }d_{i}X^{i}.$$

Also, Equations (A29)–(A36) should be transformed into

(A41) $$4{\left(X+\beta \right)}^{2}S_{r}^{2}{\left(-\frac{dW}{dX}\right)}^{2}-{\left[{\left(X+\beta \right)}^{2}-{\left(2\beta -1\right)}^{2}\right]}^{2}Q^{2}[1+{\left(-\frac{dW}{dX}\right)}^{2}]=0,$$

(A42) $${\left(X+\beta \right)}^{2}\frac{d^{2}S_{r}}{dX^{2}}+3\left(X+\beta \right)\frac{dS_{r}}{dX}+\frac{1}{2}{\left(\frac{dW}{dX}\right)}^{2}=0,$$

(A43) W=0 at X=1−β,

(A44) $$S_{r}-\nu S_{r}+(X+\beta )\frac{dS_{r}}{dX}=0\;\mathrm{at}\;X=1-\beta ,$$

(A45) $$S_{r}-\nu S_{r}+\beta \frac{dS_{r}}{dX}+X\frac{dS_{r}}{dX}=\frac{u(b)}{b}\;\mathrm{at}\;X=\beta -1,$$

(A46) $$S_{r}=\frac{1}{1-\nu }\frac{u(b)}{b}\;\mathrm{at}\;X=\beta -1,$$

(A47) $${\left(2\beta -1\right)}^{2}Q=K[W(\beta -1)-G-\bar{L}],$$

(A48) $$\begin{array}{c} \{8(X+\beta {)}^{4}S_{r}^{2}-2(X+\beta {)}^{2}[(X+\beta {)}^{2}-(2\beta -1{)}^{2}{]}^{2}Q^{2}\}\frac{d^{2}S_{r}}{dX^{2}}\\ +\{24(X+\beta {)}^{3}S_{r}^{2}-6(X+\beta )[(X+\beta {)}^{2}-(2\beta -1{)}^{2}{]}^{2}Q^{2}\}\frac{dS_{r}}{dX}\\ +[(X+\beta {)}^{2}-(2\beta -1{)}^{2}{]}^{2}Q^{2}=0 \end{array}.$$

After substituting Equation (A39) into Equation (A48), the power series coefficients *c_i_* (*i* = 2, 3, 4, …) can be expressed as polynomials with regard to *c*_0_, *c*_1_, and *β* (*β* = (1 + *α*)/2 and *α* = *b*/*a*), which can be seen in Appendix B, where *c*_0_, *c*_1_, and *β* are three undetermined constants. And after substituting Equations (A39) and (A40) into Equation (A42), the power series coefficients *d_i_* (*i* = 1, 2, 3, …) can also be expressed as polynomials with regard to *c*_0_, *c*_1_, and *β*, which can be seen in Appendix C, where the remaining coefficient *d*_0_ is a dependent undetermined constant (it depends on the undetermined constants *c*_0_, *c*_1_, and *α*).

The values of the undetermined coefficients *c*_0_, *c*_1_, *β*, and *d*_0_ can be determined by using the above boundary conditions Equations (A43) and (A44), continuous conditions Equations (A45) and (A46), and the equilibrium condition Equation (A47) of the movable electrode plate. Equations (A40), (A43), and (A47) give

(A49) $$\sum_{i=0}^{\infty }d_{i}{\left(1-\beta \right)}^{i}=0,$$

(A50) $$\sum_{i=0}^{\infty }d_{i}{\left(\beta -1\right)}^{i}=\frac{{\left(2\beta -1\right)}^{2}Q}{K}+\bar{L}+G.$$

Equation (A50) minus Equation (A49) yields

(A51) $$\sum_{i=1}^{\infty }d_{i}[{\left(\beta -1\right)}^{i}-{\left(1-\beta \right)}^{i}]=\frac{{\left(2\beta -1\right)}^{2}Q}{K}+\bar{L}+G.$$

Equations (A39) and (A44)–(A46) give

(A52) $$(1-\nu )\sum_{i=0}^{\infty }c_{i}{\left(1-\beta \right)}^{i}+\sum_{i=1}^{\infty }ic_{i}{\left(1-\beta \right)}^{i-1}=0,$$

(A53) $$(1-\nu )\sum_{i=0}^{\infty }c_{i}{\left(\beta -1\right)}^{i}+\left(2\beta -1\right)\sum_{i=1}^{\infty }ic_{i}{\left(\beta -1\right)}^{i-1}=\frac{u(b)}{b},$$

(A54) $$\sum_{i=0}^{\infty }c_{i}{\left(\beta -1\right)}^{i}=\frac{1}{1-\nu }\frac{u(b)}{b}.$$

After eliminating *u*(*b*)/*b* from Equations (A53) and (A54), it is found that

(A55) $$\sum_{i=1}^{\infty }ic_{i}{\left(\beta -1\right)}^{i-1}=0.$$

Therefore, for a concrete problem in which the values of *a*, *h*, *E*, *v*, and *q* are known beforehand, the undetermined constants *c*_0_, *c*_1_, and *β* can be determined by simultaneously solving Equations (A51), (A52), and (A55). Further, with known values for *c*_0_, *c*_1_, and *β*, the remaining dependent undetermined constant *d*_0_ can be determined by Equation (A49), and the radial displacement *u*(*b*) can be determined by Equation (A54). The problem dealt with here is thus solved analytically.

With the known undetermined constants *c*_0_, *c*_1_, *β*, and *d*_0_, all the power series coefficients *c_i_* (*i* = 2, 3, 4, …,) and *d_i_* (*i* = 1, 2, 3, …,) can be determined, thus determining all the expressions of *S_r_*, *S_t_*, and *W*. Finally, from Equations (A28), (A37), and (A38), the dimensional analytical expressions of *σ_r_* and *w* can be written as

(A56) $$\sigma_{r}=E\sum_{i=0}^{\infty }c_{i}{\left(\frac{r}{a}-\frac{a+b}{2a}\right)}^{i},$$

(A57) $$w(r)=a\sum_{i=0}^{\infty }d_{i}{\left(\frac{r}{a}-\frac{a+b}{2a}\right)}^{i}.$$

The maximum membrane deflection *w_m_* is at *r* = *b*, and thus it is given by the following, from Equation (A57):

(A58) $$w_{m}=a\sum_{i=0}^{\infty }d_{i}{\left(\frac{b-a}{2a}\right)}^{i}.$$

In addition, the maximum membrane stress *σ_m_* should be in the membrane/plate contact region of 0 ≤ *r*≤*b*. It can be seen from Equations (A21) and (A22) that in the membrane/plate contact region of 0 ≤ *r*≤*b*, the stresses and strains are both uniformly distributed, and the radial stress/strain is always equal to the circumferential stress/strain. Therefore, the maximum membrane stress *σ_m_* can be determined by Equation (A22) with the known contact radius *b* and radial displacement *u*(*b*), or given by, from Equation (A56),

(A59) $$\sigma_{m}=E\sum_{i=0}^{\infty }c_{i}{\left(\frac{b-a}{2a}\right)}^{i}$$

The accuracies of the maximum membrane deflection *w_m_* and maximum membrane stress *σ_m_* play a very important role in the numerical design and calibration of the parallel plate variable capacitor-based circular wind pressure sensor proposed here.

## Appendix B

The following are the recursive formulas for the power series coefficients *c_i_*.

$$\begin{array}{l} c_{2}=-\frac{1}{4\beta^{2}(9Q^{2}\beta^{4}-24Q^{2}\beta^{3}+22Q^{2}\beta^{2}-4\beta^{2}c_{0}^{2}-8Q^{2}\beta +Q^{2})}(54Q^{2}\beta^{5}c_{1}-144Q^{2}\beta^{4}c_{1}\\ -9Q^{2}\beta^{4}+132Q^{2}\beta^{3}c_{1}-24\beta^{3}c_{0}^{2}c_{1}+24Q^{2}\beta^{3}-48Q^{2}\beta^{2}c_{1}-22Q^{2}\beta^{2}+6Q^{2}\beta c_{1}+8Q^{2}\beta -Q^{2}) \end{array}$$

$$\begin{array}{l} c_{3}=-\frac{1}{6\beta^{2}(9Q^{2}\beta^{4}-24Q^{2}\beta^{3}+22Q^{2}\beta^{2}-4\beta^{2}c_{0}^{2}-8Q^{2}\beta +Q^{2})}(66Q^{2}\beta^{5}c_{2}-9Q^{2}\beta^{4}c_{1}\\ -208Q^{2}\beta^{4}c_{2}-16\beta^{4}c_{0}c_{1}c_{2}-24Q^{2}\beta^{3}c_{1}+212Q^{2}\beta^{3}c_{2}-56\beta^{3}c_{0}^{2}c_{2}-24\beta^{3}c_{0}c_{1}^{2}+6Q^{2}\beta^{3}\\ +54Q^{2}\beta^{2}c_{1}-80Q^{2}\beta^{2}c_{2}-36\beta^{2}c_{0}^{2}c_{1}-8Q^{2}\beta^{2}-24Q^{2}\beta c_{1}+10Q^{2}\beta c_{2}+2Q^{2}\beta +3Q^{2}c_{1}) \end{array}$$

$$\begin{array}{l} c_{4}=-\frac{1}{12\beta^{2}(9Q^{2}\beta^{4}-24Q^{2}\beta^{3}+22Q^{2}\beta^{2}-4\beta^{2}c_{0}^{2}-8Q^{2}\beta +Q^{2})}(117Q^{2}\beta^{5}c_{3}-52Q^{2}\beta^{4}c_{2}\\ -408Q^{2}\beta^{4}c_{3}-48\beta^{4}c_{0}c_{1}c_{3}-16\beta^{4}c_{0}c_{2}^{2}-8\beta^{4}c_{1}^{2}c_{2}-42Q^{2}\beta^{3}c_{1}-16Q^{2}\beta^{3}c_{2}+438Q^{2}\beta^{3}c_{3}\\ -132\beta^{3}c_{0}^{2}c_{3}-136\beta^{3}c_{0}c_{1}c_{2}-12\beta^{3}c_{1}^{3}+72Q^{2}\beta^{2}c_{1}+132Q^{2}\beta^{2}c_{2}-168Q^{2}\beta^{2}c_{3}-120\beta^{2}c_{0}^{2}c_{2}\\ -72\beta^{2}c_{0}c_{1}^{2}+Q^{2}\beta^{2}-18Q^{2}\beta c_{1}-64Q^{2}\beta c_{2}+21Q^{2}\beta c_{3}-36\beta c_{0}^{2}c_{1}-4Q^{2}\beta +8Q^{2}c_{2}+Q^{2}) \end{array}$$

$$\begin{array}{l} c_{5}=-\frac{1}{20\beta^{2}(9Q^{2}\beta^{4}-24Q^{2}\beta^{3}+22Q^{2}\beta^{2}-4\beta^{2}c_{0}^{2}-8Q^{2}\beta +Q^{2})}(180Q^{2}\beta^{5}c_{4}-129Q^{2}\beta^{4}c_{3}\\ -672Q^{2}\beta^{4}c_{4}-96\beta^{4}c_{0}c_{1}c_{4}-64\beta^{4}c_{0}c_{2}c_{3}-24\beta^{4}c_{1}^{2}c_{3}-16\beta^{4}c_{1}c_{2}^{2}-108Q^{2}\beta^{3}c_{2}+24Q^{2}\beta^{3}c_{3}\\ +744Q^{2}\beta^{3}c_{4}-240\beta^{3}c_{0}^{2}c_{4}-288\beta^{3}c_{0}c_{1}c_{3}-112\beta^{3}c_{0}c_{2}^{2}-80\beta^{3}c_{1}^{2}c_{2}+6Q^{2}\beta^{2}c_{1}+208Q^{2}\beta^{2}c_{2}\\ +234Q^{2}\beta^{2}c_{3}-288Q^{2}\beta^{2}c_{4}-252\beta^{2}c_{0}^{2}c_{3}-312\beta^{2}c_{0}c_{1}c_{2}-36\beta^{2}c_{1}^{3}+24Q^{2}\beta c_{1}-52Q^{2}\beta c_{2}\\ -120Q^{2}\beta c_{3}+36Q^{2}\beta c_{4}-104\beta c_{0}^{2}c_{2}-72\beta c_{0}c_{1}^{2}-2Q^{2}\beta -6Q^{2}c_{1}+15Q^{2}c_{3}-12c_{0}^{2}c_{1}) \end{array}$$

$$\begin{array}{l} c_{6}=-\frac{1}{60\beta^{2}(9Q^{2}\beta^{4}-24Q^{2}\beta^{3}+22Q^{2}\beta^{2}-4\beta^{2}c_{0}^{2}-8Q^{2}\beta +Q^{2})}(510Q^{2}\beta^{5}c_{5}-480Q^{2}\beta^{4}c_{4}\\ -2000Q^{2}\beta^{4}c_{5}-320\beta^{4}c_{0}c_{1}c_{5}-224\beta^{4}c_{0}c_{2}c_{4}-96\beta^{4}c_{0}c_{3}^{2}-96\beta^{4}c_{1}^{2}c_{4}-128\beta^{4}c_{1}c_{2}c_{3}-16\beta^{4}c_{2}^{3}\\ -396Q^{2}\beta^{3}c_{3}+192Q^{2}\beta^{3}c_{4}+2260Q^{2}\beta^{3}c_{5}-760\beta^{3}c_{0}^{2}c_{5}-1008\beta^{3}c_{0}c_{1}c_{4}-752\beta^{3}c_{0}c_{2}c_{3}\\ -312\beta^{3}c_{1}^{2}c_{3}-248\beta^{3}c_{1}c_{2}^{2}+52Q^{2}\beta^{2}c_{2}+816Q^{2}\beta^{2}c_{3}+720Q^{2}\beta^{2}c_{4}-880Q^{2}\beta^{2}c_{5}-864\beta^{2}c_{0}^{2}c_{4}\\ -1152\beta^{2}c_{0}c_{1}c_{3}-480\beta^{2}c_{0}c_{2}^{2}-384\beta^{2}c_{1}^{2}c_{2}+30Q^{2}\beta c_{1}+128Q^{2}\beta c_{2}-204Q^{2}\beta c_{3}-384Q^{2}\beta c_{4}\\ +110Q^{2}\beta c_{5}-408\beta c_{0}^{2}c_{3}-560\beta c_{0}c_{1}c_{2}-72\beta c_{1}^{3}-32Q^{2}c_{2}+48Q^{2}c_{4}-64c_{0}^{2}c_{2}-48c_{0}c_{1}^{2}-Q^{2}) \end{array}$$

$$\begin{array}{l} c_{7}=-\frac{1}{42\beta^{2}(9Q^{2}\beta^{4}-24Q^{2}\beta^{3}+22Q^{2}\beta^{2}-4\beta^{2}c_{0}^{2}-8Q^{2}\beta +Q^{2})}(342Q^{2}\beta^{5}c_{6}-385Q^{2}\beta^{4}c_{5}\\ -1392Q^{2}\beta^{4}c_{6}-240\beta^{4}c_{0}c_{1}c_{6}-176\beta^{4}c_{0}c_{2}c_{5}-144\beta^{4}c_{0}c_{3}c_{4}-80\beta^{4}c_{1}^{2}c_{5}-112\beta^{4}c_{1}c_{2}c_{4}\\ -48\beta^{4}c_{1}c_{3}^{2}-40\beta^{4}c_{2}^{2}c_{3}-312Q^{2}\beta^{3}c_{4}+200Q^{2}\beta^{3}c_{5}+1596Q^{2}\beta^{3}c_{6}-552\beta^{3}c_{0}^{2}c_{6}-784\beta^{3}c_{0}c_{1}c_{5}\\ -592\beta^{3}c_{0}c_{2}c_{4}-264\beta^{3}c_{0}c_{3}^{2}-264\beta^{3}c_{1}^{2}c_{4}-400\beta^{3}c_{1}c_{2}c_{3}-56\beta^{3}c_{2}^{3}+60Q^{2}\beta^{2}c_{3}+672Q^{2}\beta^{2}c_{4}\\ +510Q^{2}\beta^{2}c_{5}-624Q^{2}\beta^{2}c_{6}-660\beta^{2}c_{0}^{2}c_{5}-936\beta^{2}c_{0}c_{1}c_{4}-744\beta^{2}c_{0}c_{2}c_{3}-324\beta^{2}c_{1}^{2}c_{3}\\ -276\beta^{2}c_{1}c_{2}^{2}+42Q^{2}\beta c_{2}+120Q^{2}\beta c_{3}-168Q^{2}\beta c_{4}-280Q^{2}\beta c_{5}+78Q^{2}\beta c_{6}-336\beta c_{0}^{2}c_{4}\\ -480\beta c_{0}c_{1}c_{3}-208\beta c_{0}c_{2}^{2}-176\beta c_{1}^{2}c_{2}+3Q^{2}c_{1}-30Q^{2}c_{3}+35Q^{2}c_{5}-60c_{0}^{2}c_{3}-88c_{0}c_{1}c_{2}-12c_{1}^{3}) \end{array}$$

$$\begin{array}{l} c_{8}=-\frac{1}{56\beta^{2}(9Q^{2}\beta^{4}-24Q^{2}\beta^{3}+22Q^{2}\beta^{2}-4\beta^{2}c_{0}^{2}-8Q^{2}\beta +Q^{2})}(441Q^{2}\beta^{5}c_{7}-564Q^{2}\beta^{4}c_{6}\\ -1848Q^{2}\beta^{4}c_{7}-336\beta^{4}c_{0}c_{1}c_{7}-256\beta^{4}c_{0}c_{2}c_{6}-208\beta^{4}c_{0}c_{3}c_{5}-96\beta^{4}c_{0}c_{4}^{2}-120\beta^{4}c_{1}^{2}c_{6}\\ -176\beta^{4}c_{1}c_{2}c_{5}-144\beta^{4}c_{1}c_{3}c_{4}-64\beta^{4}c_{2}^{2}c_{4}-56\beta^{4}c_{2}c_{3}^{2}-450Q^{2}\beta^{3}c_{5}+336Q^{2}\beta^{3}c_{6}\\ +2142Q^{2}\beta^{3}c_{7}-756\beta^{3}c_{0}^{2}c_{7}-1128\beta^{3}c_{0}c_{1}c_{6}-872\beta^{3}c_{0}c_{2}c_{5}-744\beta^{3}c_{0}c_{3}c_{4}-404\beta^{3}c_{1}^{2}c_{5}\\ -616\beta^{3}c_{1}c_{2}c_{4}-276\beta^{3}c_{1}c_{3}^{2}-244\beta^{3}c_{2}^{2}c_{3}+108Q^{2}\beta^{2}c_{4}+1000Q^{2}\beta^{2}c_{5}+684Q^{2}\beta^{2}c_{6}\\ -840Q^{2}\beta^{2}c_{7}-936\beta^{2}c_{0}^{2}c_{6}-1392\beta^{2}c_{0}c_{1}c_{5}-1104\beta^{2}c_{0}c_{2}c_{4}-504\beta^{2}c_{0}c_{3}^{2}-504\beta^{2}c_{1}^{2}c_{4}\\ -816\beta^{2}c_{1}c_{2}c_{3}-120\beta^{2}c_{2}^{3}+81Q^{2}\beta c_{3}+192Q^{2}\beta c_{4}-250Q^{2}\beta c_{5}-384Q^{2}\beta c_{6}+105Q^{2}\beta c_{7}\\ -500\beta c_{0}^{2}c_{5}-744\beta c_{0}c_{1}c_{4}-616\beta c_{0}c_{2}c_{3}-276\beta c_{1}^{2}c_{3}-244\beta c_{1}c_{2}^{2}+8Q^{2}c_{2}-48Q^{2}c_{4}+48Q^{2}c_{6}\\ -96c_{0}^{2}c_{4}-144c_{0}c_{1}c_{3}-64c_{0}c_{2}^{2}-56c_{1}^{2}c_{2}) \end{array}$$

## Appendix C

The following are the recursive formulas for the power series coefficients *d_i_*.

$$d_{1}=\frac{(\beta -1)(3\beta -1)Q}{\sqrt{-9Q^{2}\beta^{4}+24Q^{2}\beta^{3}-22Q^{2}\beta^{2}+4\beta^{2}c_{0}^{2}+8Q^{2}\beta -Q^{2}}}$$

$$\begin{array}{l} d_{2}=\frac{1}{d_{1}(9Q^{2}\beta^{4}-24Q^{2}\beta^{3}+22Q^{2}\beta^{2}-4\beta^{2}c_{0}^{2}-8Q^{2}\beta +Q^{2})}(3Q^{2}\beta^{3}d_{1}^{2}-4Q^{2}\beta^{2}d_{1}^{2}+2\beta^{2}c_{0}c_{1}d_{1}^{2}+3Q^{2}\beta^{3}\\ +Q^{2}\beta d_{1}^{2}+2\beta c_{0}^{2}d_{1}^{2}-4Q^{2}\beta^{2}+Q^{2}\beta ) \end{array}$$

$$\begin{array}{l} d_{3}=-\frac{1}{3d_{1}(9Q^{2}\beta^{4}-24Q^{2}\beta^{3}+22Q^{2}\beta^{2}-4\beta^{2}c_{0}^{2}-8Q^{2}\beta +Q^{2})}(18Q^{2}\beta^{4}d_{2}^{2}-24Q^{2}\beta^{3}d_{1}d_{2}-48Q^{2}\beta^{3}d_{2}^{2}\\ -Q^{2}\beta^{2}d_{1}^{2}+32Q^{2}\beta^{2}d_{1}d_{2}+44Q^{2}\beta^{2}d_{2}^{2}-8\beta^{2}c_{0}^{2}d_{2}^{2}-16\beta^{2}c_{0}c_{1}d_{1}d_{2}-4\beta^{2}c_{0}c_{2}d_{1}^{2}-2\beta^{2}c_{1}^{2}d_{1}^{2}+4Q^{2}\beta d_{1}^{2}\\ -8Q^{2}\beta d_{1}d_{2}-16Q^{2}\beta d_{2}^{2}-16\beta c_{0}^{2}d_{1}d_{2}-8\beta c_{0}c_{1}d_{1}^{2}-Q^{2}\beta^{2}-Q^{2}d_{1}^{2}+2Q^{2}d_{2}^{2}-2c_{0}^{2}d_{1}^{2}+4Q^{2}\beta -Q^{2}) \end{array}$$

$$\begin{array}{l} d_{4}=-\frac{1}{2d_{1}(9Q^{2}\beta^{4}-24Q^{2}\beta^{3}+22Q^{2}\beta^{2}-4\beta^{2}c_{0}^{2}-8Q^{2}\beta +Q^{2})}(27Q^{2}\beta^{4}d_{2}d_{3}-18Q^{2}\beta^{3}d_{1}d_{3}-12Q^{2}\beta^{3}d_{2}^{2}\\ -72Q^{2}\beta^{3}d_{2}d_{3}-2Q^{2}\beta^{2}d_{1}d_{2}+24Q^{2}\beta^{2}d_{1}d_{3}+16Q^{2}\beta^{2}d_{2}^{2}+66Q^{2}\beta^{2}d_{2}d_{3}-12\beta^{2}c_{0}^{2}d_{2}d_{3}-12\beta^{2}c_{0}c_{1}d_{1}d_{3}\\ -8\beta^{2}c_{0}c_{1}d_{2}^{2}-8\beta^{2}c_{0}c_{2}d_{1}d_{2}-2\beta^{2}c_{0}c_{3}d_{1}^{2}-4\beta^{2}c_{1}^{2}d_{1}d_{2}-2\beta^{2}c_{1}c_{2}d_{1}^{2}+Q^{2}\beta d_{1}^{2}+8Q^{2}\beta d_{1}d_{2}-6Q^{2}\beta d_{1}d_{3}\\ -4Q^{2}\beta d_{2}^{2}-24Q^{2}\beta d_{2}d_{3}-12\beta c_{0}^{2}d_{1}d_{3}-8\beta c_{0}^{2}d_{2}^{2}-16\beta c_{0}c_{1}d_{1}d_{2}-4\beta c_{0}c_{2}d_{1}^{2}-2\beta c_{1}^{2}d_{1}^{2}-2Q^{2}d_{1}d_{2}+3Q^{2}d_{2}d_{3}\\ -4c_{0}^{2}d_{1}d_{2}-2c_{0}c_{1}d_{1}^{2}+Q^{2}\beta ) \end{array}$$

$$\begin{array}{l} d_{5}=-\frac{1}{10d_{1}(9Q^{2}\beta^{4}-24Q^{2}\beta^{3}+22Q^{2}\beta^{2}-4\beta^{2}c_{0}^{2}-8Q^{2}\beta +Q^{2})}(144Q^{2}\beta^{4}d_{2}d_{4}+81Q^{2}\beta^{4}d_{3}^{2}-96Q^{2}\beta^{3}d_{1}d_{4}\\ -144Q^{2}\beta^{3}d_{2}d_{3}-384Q^{2}\beta^{3}d_{2}d_{4}-216Q^{2}\beta^{3}d_{3}^{2}-12Q^{2}\beta^{2}d_{1}d_{3}+128Q^{2}\beta^{2}d_{1}d_{4}-8Q^{2}\beta^{2}d_{2}^{2}+192Q^{2}\beta^{2}d_{2}d_{3}\\ +352Q^{2}\beta^{2}d_{2}d_{4}+198Q^{2}\beta^{2}d_{3}^{2}-64\beta^{2}c_{0}^{2}d_{2}d_{4}-36\beta^{2}c_{0}^{2}d_{3}^{2}-64\beta^{2}c_{0}c_{1}d_{1}d_{4}-96\beta^{2}c_{0}c_{1}d_{2}d_{3}-48\beta^{2}c_{0}c_{2}d_{1}d_{3}\\ -32\beta^{2}c_{0}c_{2}d_{2}^{2}-32\beta^{2}c_{0}c_{3}d_{1}d_{2}-8\beta^{2}c_{0}c_{4}d_{1}^{2}-24\beta^{2}c_{1}^{2}d_{1}d_{3}-16\beta^{2}c_{1}^{2}d_{2}^{2}-32\beta^{2}c_{1}c_{2}d_{1}d_{2}-8\beta^{2}c_{1}c_{3}d_{1}^{2}\\ -4\beta^{2}c_{2}^{2}d_{1}^{2}+16Q^{2}\beta d_{1}d_{2}+48Q^{2}\beta d_{1}d_{3}-32Q^{2}\beta d_{1}d_{4}+32Q^{2}\beta d_{2}^{2}-48Q^{2}\beta d_{2}d_{3}-128Q^{2}\beta d_{2}d_{4}-72Q^{2}\beta d_{3}^{2}\\ -64\beta c_{0}^{2}d_{1}d_{4}-96\beta c_{0}^{2}d_{2}d_{3}-96\beta c_{0}c_{1}d_{1}d_{3}-64\beta c_{0}c_{1}d_{2}^{2}-64\beta c_{0}c_{2}d_{1}d_{2}-16\beta c_{0}c_{3}d_{1}^{2}-32\beta c_{1}^{2}d_{1}d_{2}-16\beta c_{1}c_{2}d_{1}^{2}\\ +Q^{2}d_{1}^{2}+Q^{2}-12Q^{2}d_{1}d_{3}-8Q^{2}d_{2}^{2}+16Q^{2}d_{2}d_{4}+9Q^{2}d_{3}^{2}-24c_{0}^{2}d_{1}d_{3}-16c_{0}^{2}d_{2}^{2}-32c_{0}c_{1}d_{1}d_{2}-8c_{0}c_{2}d_{1}^{2}-4c_{1}^{2}d_{1}^{2}) \end{array}$$

$$\begin{array}{l} d_{6}=-\frac{1}{3d_{1}(9Q^{2}\beta^{4}-24Q^{2}\beta^{3}+22Q^{2}\beta^{2}-4\beta^{2}c_{0}^{2}-8Q^{2}\beta +Q^{2})}(45Q^{2}\beta^{4}d_{2}d_{5}+54Q^{2}\beta^{4}d_{3}d_{4}-30Q^{2}\beta^{3}d_{1}d_{5}\\ -48Q^{2}\beta^{3}d_{2}d_{4}-120Q^{2}\beta^{3}d_{2}d_{5}-27Q^{2}\beta^{3}d_{3}^{2}-144Q^{2}\beta^{3}d_{3}d_{4}-4Q^{2}\beta^{2}d_{1}d_{4}+40Q^{2}\beta^{2}d_{1}d_{5}-6Q^{2}\beta^{2}d_{2}d_{3}+64Q^{2}\beta^{2}d_{2}d_{4}\\ +110Q^{2}\beta^{2}d_{2}d_{5}+36Q^{2}\beta^{2}d_{3}^{2}+132Q^{2}\beta^{2}d_{3}d_{4}-20\beta^{2}c_{0}^{2}d_{2}d_{5}-24\beta^{2}c_{0}^{2}d_{3}d_{4}-20\beta^{2}c_{0}c_{1}d_{1}d_{5}-32\beta^{2}c_{0}c_{1}d_{2}d_{4}\\ -18\beta^{2}c_{0}c_{1}d_{3}^{2}-16\beta^{2}c_{0}c_{2}d_{1}d_{4}-24\beta^{2}c_{0}c_{2}d_{2}d_{3}-12\beta^{2}c_{0}c_{3}d_{1}d_{3}-8\beta^{2}c_{0}c_{3}d_{2}^{2}-8\beta^{2}c_{0}c_{4}d_{1}d_{2}-2\beta^{2}c_{0}c_{5}d_{1}^{2}-8\beta^{2}c_{1}^{2}d_{1}d_{4}\\ -12\beta^{2}c_{1}^{2}d_{2}d_{3}-12\beta^{2}c_{1}c_{2}d_{1}d_{3}-8\beta^{2}c_{1}c_{2}d_{2}^{2}-8\beta^{2}c_{1}c_{3}d_{1}d_{2}-2\beta^{2}c_{1}c_{4}d_{1}^{2}-4\beta^{2}c_{2}^{2}d_{1}d_{2}-2\beta^{2}c_{2}c_{3}d_{1}^{2}+6Q^{2}\beta d_{1}d_{3}\\ +16Q^{2}\beta d_{1}d_{4}-10Q^{2}\beta d_{1}d_{5}+4Q^{2}\beta d_{2}^{2}+24Q^{2}\beta d_{2}d_{3}-16Q^{2}\beta d_{2}d_{4}-40Q^{2}\beta d_{2}d_{5}-9Q^{2}\beta d_{3}^{2}-48Q^{2}\beta d_{3}d_{4}-20\beta c_{0}^{2}d_{1}d_{5}\\ -32\beta c_{0}^{2}d_{2}d_{4}-18\beta c_{0}^{2}d_{3}^{2}-32\beta c_{0}c_{1}d_{1}d_{4}-48\beta c_{0}c_{1}d_{2}d_{3}-24\beta c_{0}c_{2}d_{1}d_{3}-16\beta c_{0}c_{2}d_{2}^{2}-16\beta c_{0}c_{3}d_{1}d_{2}-4\beta c_{0}c_{4}d_{1}^{2}\\ -12\beta c_{1}^{2}d_{1}d_{3}-8\beta c_{1}^{2}d_{2}^{2}-16\beta c_{1}c_{2}d_{1}d_{2}-4\beta c_{1}c_{3}d_{1}^{2}-2\beta c_{2}^{2}d_{1}^{2}+Q^{2}d_{1}d_{2}-4Q^{2}d_{1}d_{4}-6Q^{2}d_{2}d_{3}+5Q^{2}d_{2}d_{5}+6Q^{2}d_{3}d_{4}\\ -8c_{0}^{2}d_{1}d_{4}-12c_{0}^{2}d_{2}d_{3}-12c_{0}c_{1}d_{1}d_{3}-8c_{0}c_{1}d_{2}^{2}-8c_{0}c_{2}d_{1}d_{2}-2c_{0}c_{3}d_{1}^{2}-4c_{1}^{2}d_{1}d_{2}-2c_{1}c_{2}d_{1}^{2}) \end{array}$$

$$\begin{array}{l} d_{7}=-\frac{1}{7d_{1}(9Q^{2}\beta^{4}-24Q^{2}\beta^{3}+22Q^{2}\beta^{2}-4\beta^{2}c_{0}^{2}-8Q^{2}\beta +Q^{2})}(108Q^{2}\beta^{4}d_{2}d_{6}+135Q^{2}\beta^{4}d_{3}d_{5}+72Q^{2}\beta^{4}d_{4}^{2}\\ -72Q^{2}\beta^{3}d_{1}d_{6}-120Q^{2}\beta^{3}d_{2}d_{5}-288Q^{2}\beta^{3}d_{2}d_{6}-144Q^{2}\beta^{3}d_{3}d_{4}-360Q^{2}\beta^{3}d_{3}d_{5}-192Q^{2}\beta^{3}d_{4}^{2}-10Q^{2}\beta^{2}d_{1}d_{5}\\ +96Q^{2}\beta^{2}d_{1}d_{6}-16Q^{2}\beta^{2}d_{2}d_{4}+160Q^{2}\beta^{2}d_{2}d_{5}+264Q^{2}\beta^{2}d_{2}d_{6}-9Q^{2}\beta^{2}d_{3}^{2}+192Q^{2}\beta^{2}d_{3}d_{4}+330Q^{2}\beta^{2}d_{3}d_{5}\\ +176Q^{2}\beta^{2}d_{4}^{2}-48\beta^{2}c_{0}^{2}d_{2}d_{6}-60\beta^{2}c_{0}^{2}d_{3}d_{5}-32\beta^{2}c_{0}^{2}d_{4}^{2}-48\beta^{2}c_{0}c_{1}d_{1}d_{6}-80\beta^{2}c_{0}c_{1}d_{2}d_{5}-96\beta^{2}c_{0}c_{1}d_{3}d_{4}-40\beta^{2}c_{0}c_{2}d_{1}d_{5}\\ -64\beta^{2}c_{0}c_{2}d_{2}d_{4}-36\beta^{2}c_{0}c_{2}d_{3}^{2}-32\beta^{2}c_{0}c_{3}d_{1}d_{4}-48\beta^{2}c_{0}c_{3}d_{2}d_{3}-24\beta^{2}c_{0}c_{4}d_{1}d_{3}-16\beta^{2}c_{0}c_{4}d_{2}^{2}-16\beta^{2}c_{0}c_{5}d_{1}d_{2}\\ -4\beta^{2}c_{0}c_{6}d_{1}^{2}-20\beta^{2}c_{1}^{2}d_{1}d_{5}-32\beta^{2}c_{1}^{2}d_{2}d_{4}-18\beta^{2}c_{1}^{2}d_{3}^{2}-32\beta^{2}c_{1}c_{2}d_{1}d_{4}-48\beta^{2}c_{1}c_{2}d_{2}d_{3}-24\beta^{2}c_{1}c_{3}d_{1}d_{3}-16\beta^{2}c_{1}c_{3}d_{2}^{2}\\ -16\beta^{2}c_{1}c_{4}d_{1}d_{2}-4\beta^{2}c_{1}c_{5}d_{1}^{2}-12\beta^{2}c_{2}^{2}d_{1}d_{3}-8\beta^{2}c_{2}^{2}d_{2}^{2}-16\beta^{2}c_{2}c_{3}d_{1}d_{2}-4\beta^{2}c_{2}c_{4}d_{1}^{2}-2\beta^{2}c_{3}^{2}d_{1}^{2}+16Q^{2}\beta d_{1}d_{4}\\ +40Q^{2}\beta d_{1}d_{5}-24Q^{2}\beta d_{1}d_{6}+24Q^{2}\beta d_{2}d_{3}+64Q^{2}\beta d_{2}d_{4}-40Q^{2}\beta d_{2}d_{5}-96Q^{2}\beta d_{2}d_{6}+36Q^{2}\beta d_{3}^{2}-48Q^{2}\beta d_{3}d_{4}\\ -120Q^{2}\beta d_{3}d_{5}-64Q^{2}\beta d_{4}^{2}-48\beta c_{0}^{2}d_{1}d_{6}-80\beta c_{0}^{2}d_{2}d_{5}-96\beta c_{0}^{2}d_{3}d_{4}-80\beta c_{0}c_{1}d_{1}d_{5}-128\beta c_{0}c_{1}d_{2}d_{4}-72\beta c_{0}c_{1}d_{3}^{2}\\ -64\beta c_{0}c_{2}d_{1}d_{4}-96\beta c_{0}c_{2}d_{2}d_{3}-48\beta c_{0}c_{3}d_{1}d_{3}-32\beta c_{0}c_{3}d_{2}^{2}-32\beta c_{0}c_{4}d_{1}d_{2}-8\beta c_{0}c_{5}d_{1}^{2}-32\beta c_{1}^{2}d_{1}d_{4}-48\beta c_{1}^{2}d_{2}d_{3}\\ -48\beta c_{1}c_{2}d_{1}d_{3}-32\beta c_{1}c_{2}d_{2}^{2}-32\beta c_{1}c_{3}d_{1}d_{2}-8\beta c_{1}c_{4}d_{1}^{2}-16\beta c_{2}^{2}d_{1}d_{2}-8\beta c_{2}c_{3}d_{1}^{2}+3Q^{2}d_{1}d_{3}-10Q^{2}d_{1}d_{5}+2Q^{2}d_{2}^{2}\\ -16Q^{2}d_{2}d_{4}+12Q^{2}d_{2}d_{6}-9Q^{2}d_{3}^{2}+15Q^{2}d_{3}d_{5}+8Q^{2}d_{4}^{2}-20c_{0}^{2}d_{1}d_{5}-32c_{0}^{2}d_{2}d_{4}-18c_{0}^{2}d_{3}^{2}-32c_{0}c_{1}d_{1}d_{4}-48c_{0}c_{1}d_{2}d_{3}\\ -24c_{0}c_{2}d_{1}d_{3}-16c_{0}c_{2}d_{2}^{2}-16c_{0}c_{3}d_{1}d_{2}-4c_{0}c_{4}d_{1}^{2}-12c_{1}^{2}d_{1}d_{3}-8c_{1}^{2}d_{2}^{2}-16c_{1}c_{2}d_{1}d_{2}-4c_{1}c_{3}d_{1}^{2}-2c_{2}^{2}d_{1}^{2}) \end{array}$$

$$\begin{array}{l} d_{8}=-\frac{1}{4d_{1}(9Q^{2}\beta^{4}-24Q^{2}\beta^{3}+22Q^{2}\beta^{2}-4\beta^{2}c_{0}^{2}-8Q^{2}\beta +Q^{2})}(63Q^{2}\beta^{4}d_{2}d_{7}+81Q^{2}\beta^{4}d_{3}d_{6}+90Q^{2}\beta^{4}d_{4}d_{5}\\ -42Q^{2}\beta^{3}d_{1}d_{7}-72Q^{2}\beta^{3}d_{2}d_{6}-168Q^{2}\beta^{3}d_{2}d_{7}-90Q^{2}\beta^{3}d_{3}d_{5}-216Q^{2}\beta^{3}d_{3}d_{6}-48Q^{2}\beta^{3}d_{4}^{2}-240Q^{2}\beta^{3}d_{4}d_{5}-6Q^{2}\beta^{2}d_{1}d_{6}\\ +56Q^{2}\beta^{2}d_{1}d_{7}-10Q^{2}\beta^{2}d_{2}d_{5}+96Q^{2}\beta^{2}d_{2}d_{6}+154Q^{2}\beta^{2}d_{2}d_{7}-12Q^{2}\beta^{2}d_{3}d_{4}+120Q^{2}\beta^{2}d_{3}d_{5}+198Q^{2}\beta^{2}d_{3}d_{6}+64Q^{2}\beta^{2}d_{4}^{2}\\ +220Q^{2}\beta^{2}d_{4}d_{5}-28\beta^{2}c_{0}^{2}d_{2}d_{7}-36\beta^{2}c_{0}^{2}d_{3}d_{6}-40\beta^{2}c_{0}^{2}d_{4}d_{5}-28\beta^{2}c_{0}c_{1}d_{1}d_{7}-48\beta^{2}c_{0}c_{1}d_{2}d_{6}-60\beta^{2}c_{0}c_{1}d_{3}d_{5}-32\beta^{2}c_{0}c_{1}d_{4}^{2}\\ -24\beta^{2}c_{0}c_{2}d_{1}d_{6}-40\beta^{2}c_{0}c_{2}d_{2}d_{5}-48\beta^{2}c_{0}c_{2}d_{3}d_{4}-20\beta^{2}c_{0}c_{3}d_{1}d_{5}-32\beta^{2}c_{0}c_{3}d_{2}d_{4}-18\beta^{2}c_{0}c_{3}d_{3}^{2}-16\beta^{2}c_{0}c_{4}d_{1}d_{4}\\ -24\beta^{2}c_{0}c_{4}d_{2}d_{3}-12\beta^{2}c_{0}c_{5}d_{1}d_{3}-8\beta^{2}c_{0}c_{5}d_{2}^{2}-8\beta^{2}c_{0}c_{6}d_{1}d_{2}-2\beta^{2}c_{0}c_{7}d_{1}^{2}-12\beta^{2}c_{1}^{2}d_{1}d_{6}-20\beta^{2}c_{1}^{2}d_{2}d_{5}-24\beta^{2}c_{1}^{2}d_{3}d_{4}\\ -20\beta^{2}c_{1}c_{2}d_{1}d_{5}-32\beta^{2}c_{1}c_{2}d_{2}d_{4}-18\beta^{2}c_{1}c_{2}d_{3}^{2}-16\beta^{2}c_{1}c_{3}d_{1}d_{4}-24\beta^{2}c_{1}c_{3}d_{2}d_{3}-12\beta^{2}c_{1}c_{4}d_{1}d_{3}-8\beta^{2}c_{1}c_{4}d_{2}^{2}-8\beta^{2}c_{1}c_{5}d_{1}d_{2}\\ -2\beta^{2}c_{1}c_{6}d_{1}^{2}-8\beta^{2}c_{2}^{2}d_{1}d_{4}-12\beta^{2}c_{2}^{2}d_{2}d_{3}-12\beta^{2}c_{2}c_{3}d_{1}d_{3}-8\beta^{2}c_{2}c_{3}d_{2}^{2}-8\beta^{2}c_{2}c_{4}d_{1}d_{2}-2\beta^{2}c_{2}c_{5}d_{1}^{2}-4\beta^{2}c_{3}^{2}d_{1}d_{2}-2\beta^{2}c_{3}c_{4}d_{1}^{2}\\ +10Q^{2}\beta d_{1}d_{5}+24Q^{2}\beta d_{1}d_{6}-14Q^{2}\beta d_{1}d_{7}+16Q^{2}\beta d_{2}d_{4}+40Q^{2}\beta d_{2}d_{5}-24Q^{2}\beta d_{2}d_{6}-56Q^{2}\beta d_{2}d_{7}+9Q^{2}\beta d_{3}^{2}+48Q^{2}\beta d_{3}d_{4}\\ -30Q^{2}\beta d_{3}d_{5}-72Q^{2}\beta d_{3}d_{6}-16Q^{2}\beta d_{4}^{2}-80Q^{2}\beta d_{4}d_{5}-28\beta c_{0}^{2}d_{1}d_{7}-48\beta c_{0}^{2}d_{2}d_{6}-60\beta c_{0}^{2}d_{3}d_{5}-32\beta c_{0}^{2}d_{4}^{2}-48\beta c_{0}c_{1}d_{1}d_{6}\\ -80\beta c_{0}c_{1}d_{2}d_{5}-96\beta c_{0}c_{1}d_{3}d_{4}-40\beta c_{0}c_{2}d_{1}d_{5}-64\beta c_{0}c_{2}d_{2}d_{4}-36\beta c_{0}c_{2}d_{3}^{2}-32\beta c_{0}c_{3}d_{1}d_{4}-48\beta c_{0}c_{3}d_{2}d_{3}-24\beta c_{0}c_{4}d_{1}d_{3}\\ -16\beta c_{0}c_{4}d_{2}^{2}-16\beta c_{0}c_{5}d_{1}d_{2}-4\beta c_{0}c_{6}d_{1}^{2}-20\beta c_{1}^{2}d_{1}d_{5}-32\beta c_{1}^{2}d_{2}d_{4}-18\beta c_{1}^{2}d_{3}^{2}-32\beta c_{1}c_{2}d_{1}d_{4}-48\beta c_{1}c_{2}d_{2}d_{3}-24\beta c_{1}c_{3}d_{1}d_{3}\\ -16\beta c_{1}c_{3}d_{2}^{2}-16\beta c_{1}c_{4}d_{1}d_{2}-4\beta c_{1}c_{5}d_{1}^{2}-12\beta c_{2}^{2}d_{1}d_{3}-8\beta c_{2}^{2}d_{2}^{2}-16\beta c_{2}c_{3}d_{1}d_{2}-4\beta c_{2}c_{4}d_{1}^{2}-2\beta c_{3}^{2}d_{1}^{2}+2Q^{2}d_{1}d_{4}-6Q^{2}d_{1}d_{6}\\ +3Q^{2}d_{2}d_{3}-10Q^{2}d_{2}d_{5}+7Q^{2}d_{2}d_{7}-12Q^{2}d_{3}d_{4}+9Q^{2}d_{3}d_{6}+10Q^{2}d_{4}d_{5}-12c_{0}^{2}d_{1}d_{6}-20c_{0}^{2}d_{2}d_{5}-24c_{0}^{2}d_{3}d_{4}-20c_{0}c_{1}d_{1}d_{5}\\ -32c_{0}c_{1}d_{2}d_{4}-18c_{0}c_{1}d_{3}^{2}-16c_{0}c_{2}d_{1}d_{4}-24c_{0}c_{2}d_{2}d_{3}-12c_{0}c_{3}d_{1}d_{3}-8c_{0}c_{3}d_{2}^{2}-8c_{0}c_{4}d_{1}d_{2}-2c_{0}c_{5}d_{1}^{2}-8c_{1}^{2}d_{1}d_{4}-12c_{1}^{2}d_{2}d_{3}\\ -12c_{1}c_{2}d_{1}d_{3}-8c_{1}c_{2}d_{2}^{2}-8c_{1}c_{3}d_{1}d_{2}-2c_{1}c_{4}d_{1}^{2}-4c_{2}^{2}d_{1}d_{2}-2c_{2}c_{3}d_{1}^{2}) \end{array}$$

## Appendix D

The following are the results of the numerical calculations in Section 3.2 and Section 3.3.

**Table A1:** The numerical calculation results of the undetermined constants *β*, *c*_0_, *c*_1_, and *d*_0_ as well as maximum stress *σ_m_* and maximum deflection *w_m_* when *k* = 0.5 N/mm, *a* = 70 mm, *h* = 0.3 mm, *t* = 0.1 mm, *E* = 3.01 MPa, *ν* = 0.45, *g* = 5 mm, Δ*l* = 5 mm, *L* = 40 mm, and the wind pressure *q* takes different values.

*q*/Pa	*β*	*c*_0_	*c*_1_	*d*_0_	*σ_m_/*MPa	*w_m_*/mm	*C/*pF
164	0.508089	0.023938	−0.004382	0.109411	0.075231	10.00132	3.8923
180	0.544050	0.024999	−0.004715	0.107564	0.078310	10.0430	3.8969
250	0.598700	0.029582	−0.005228	0.109212	0.091588	10.2999	3.9257
450	0.649548	0.040977	−0.006322	0.119276	0.125661	11.2394	4.0349
650	0.666143	0.051272	−0.007420	0.129987	0.156822	12.2096	4.1541
1000	0.675506	0.068129	−0.009289	0.147594	0.208037	13.7933	4.3647
2000	0.674910	0.112451	−0.014101	0.189765	0.342976	17.5352	4.9585
3000	0.668757	0.153851	−0.018268	0.224077	0.469091	20.5216	5.5626
4000	0.662773	0.193468	−0.021953	0.253639	0.590176	23.0514	6.2026
5000	0.657493	0.232254	−0.025264	0.279991	0.707882	25.2732	6.8999
6000	0.653780	0.269163	−0.028209	0.304693	0.825293	27.3276	7.7003
7000	0.649841	0.307092	−0.031039	0.326136	0.939422	29.0912	8.5520
8000	0.645980	0.344476	−0.033645	0.347378	1.051860	30.8186	9.5910
9000	0.642338	0.381389	−0.036051	0.367314	1.156710	32.4215	10.8096
10000	0.639188	0.416372	−0.038191	0.385375	1.268142	33.8582	12.1988
11000	0.636385	0.452081	−0.040249	0.403105	1.375390	35.2552	13.9410
12000	0.633941	0.488016	−0.042216	0.420358	1.483440	36.6031	16.1690
13000	0.631907	0.521772	−0.043989	0.436153	1.590910	37.8286	18.9179
14000	0.629948	0.557385	−0.045807	0.452525	1.697980	39.0928	22.9413
15000	0.628117	0.592392	−0.047568	0.468470	1.803200	40.3209	28.9153

**Table A2:** The numerical calculation results of the undetermined constants *β*, *c*_0_, *c*_1_, and *d*_0_ as well as maximum stress *σ_m_* and maximum deflection *w_m_* when *k* = 0.1 N/mm, *a* = 70 mm, *h* = 0.3 mm, *t* = 0.1 mm, *E* = 3.01 MPa, *ν* = 0.45, *g* = 5 mm, Δ*l* = 5 mm, *L* = 40 mm, and the wind pressure *q* takes different values.

*q*/Pa	*β*	*c*_0_	*c_1_*	*d*_0_	*σ_m_/*MPa	*w_m_*/mm	*C*/pF
164	0.506914	0.023946	−0.004374	0.109573	0.075252	10.00483	3.8927
800	0.602296	0.065844	−0.010839	0.162026	0.203325	15.1548	4.5635
1600	0.597546	0.108481	−0.016790	0.209132	0.334643	19.3744	5.3139
2400	0.591984	0.146778	−0.021637	0.244847	0.452539	22.5038	6.0519
3200	0.587431	0.182701	−0.025776	0.274563	0.563003	25.0620	6.8270
4000	0.583712	0.217076	−0.029406	0.300480	0.668585	27.2600	7.6711
4800	0.580613	0.250345	−0.032648	0.323739	0.770656	29.2067	8.6144
5600	0.577964	0.282437	−0.035552	0.344809	0.869017	30.9494	9.6800
6400	0.575697	0.314539	−0.038259	0.364750	0.967315	32.5811	10.9481
7200	0.573715	0.345632	−0.040715	0.383163	1.062450	34.0728	12.4376
8000	0.571922	0.376531	−0.043012	0.400710	1.156913	35.4815	14.2712
8800	0.570320	0.406999	−0.045152	0.417383	1.250000	36.8088	16.5733
9600	0.568894	0.436963	−0.047137	0.433192	1.341495	38.0567	19.5361
10400	0.567597	0.466929	−0.049006	0.448450	1.432930	39.2508	23.5677
11200	0.566406	0.496490	−0.050768	0.463113	1.523100	40.3908	29.3503

**Table A3:** The numerical calculation results of the undetermined constants *β*, *c*_0_, *c*_1_, and *d*_0_ as well as maximum stress *σ_m_* and maximum deflection *w_m_* when *k* = 1 N/mm, *a* = 70 mm, *h* = 0.3 mm, *t* = 0.1 mm, *E* = 3.01 MPa, *ν* = 0.45, *g* = 5 mm, Δ*l* = 5 mm, *L* = 40 mm, and the wind pressure *q* takes different values.

*q*/Pa	*β*	*c*_0_	*c_1_*	*d*_0_	*σ_m_/*MPa	*w_m_*/mm	*C*/pF
164	0.508286	0.023936	−0.004383	0.109384	0.075227	10.00069	3.8922
1300	0.711565	0.075547	−0.009120	0.146106	0.229843	13.5829	4.3355
2600	0.712401	0.125259	−0.013597	0.187818	0.380656	17.2226	4.9028
3900	0.706387	0.172868	−0.017515	0.222950	0.525154	20.2291	5.4970
5200	0.700146	0.219350	−0.021007	0.253841	0.666239	22.8264	6.1398
6500	0.694480	0.265086	−0.024166	0.281772	0.805056	25.1381	6.8531
7800	0.689450	0.310275	−0.027056	0.307505	0.942204	27.2381	7.6616
9100	0.684909	0.355158	−0.029732	0.331608	1.078420	29.1799	8.5998
10400	0.680995	0.399444	−0.032206	0.354198	1.212813	30.9785	9.7001
11700	0.677361	0.444432	−0.034572	0.376153	1.349330	32.7074	11.0602
13000	0.674166	0.487414	−0.036712	0.396342	1.479763	34.2815	12.6789
14300	0.671252	0.530229	−0.038741	0.415799	1.609680	35.7850	14.7393
15600	0.668504	0.574475	−0.040732	0.435253	1.743941	37.2741	17.5666
16900	0.666055	0.617610	−0.042573	0.453884	1.874752	38.6875	21.4770
18200	0.663750	0.661042	−0.044355	0.471687	2.006610	40.0263	27.2152

**Table A4:** The numerical calculation results of the undetermined constants *β*, *c*_0_, *c*_1_, and *d*_0_ as well as maximum stress *σ_m_* and maximum deflection *w_m_* when *k* = 0.5 N/mm, *a* = 80 mm, *h* = 0.3 mm, *t* = 0.1 mm, *E* = 3.01 MPa, *ν* = 0.45, *g* = 5 mm, Δ*l* = 5 mm, *L* = 40 mm, and the wind pressure *q* takes different values.

*q*/Pa	*β*	*c*_0_	*c_1_*	*d*_0_	*σ_m_/*MPa	*w_m_*/mm	*C*/pF
96	0.503799	0.018254	−0.003345	0.096033	0.057379	10.00022	5.0836
700	0.689550	0.056414	−0.007571	0.131199	0.172069	14.0454	5.7472
1400	0.687513	0.092996	−0.011650	0.169036	0.283418	17.9179	6.5679
2100	0.680619	0.127106	−0.015243	0.199852	0.387316	21.0196	7.4160
2800	0.674104	0.159795	−0.018466	0.226400	0.486893	23.6520	8.3289
3500	0.668405	0.191525	−0.021395	0.250050	0.583536	25.9660	9.3395
4200	0.663445	0.222550	−0.024083	0.271581	0.678017	28.0474	10.4837
4900	0.659001	0.253292	−0.026592	0.291659	0.771615	29.9668	11.8189
5600	0.655252	0.283073	−0.028888	0.310091	0.862270	31.7111	13.3660
6300	0.651754	0.313475	−0.031107	0.328048	0.954795	33.3943	15.2983
7000	0.648721	0.342126	−0.033096	0.344292	1.041976	34.9036	17.5769
7700	0.645941	0.372417	−0.034996	0.359974	1.129190	36.3493	20.5018
8400	0.643347	0.400114	−0.036845	0.375443	1.218373	37.7635	24.4880
9100	0.641027	0.428760	−0.038560	0.390028	1.305490	39.0855	29.9274
9800	0.638853	0.457456	−0.040213	0.404264	1.392740	40.3676	38.1446
10500	0.636795	0.485605	−0.041803	0.418045	1.478328	41.6044	51.8883

**Table A5:** The numerical calculation results of the undetermined constants *β*, *c*_0_, *c*_1_, and *d*_0_ as well as maximum stress *σ_m_* and maximum deflection *w_m_* when *k* = 0.5 N/mm, *a* = 90 mm, *h* = 0.3 mm, *t* = 0.1 mm, *E* = 3.01 MPa, *ν* = 0.45, *g* = 5 mm, Δ*l* = 5 mm, *L* = 40 mm, and the wind pressure *q* takes different values.

*q*/Pa	*β*	*c*_0_	*c_1_*	*d*_0_	*σ_m_/*MPa	*w_m_*/mm	*C*/pF
60	0.503156	0.014394	−0.002651	0.085357	0.045259	10.00012	6.4340
450	0.700743	0.043775	−0.005850	0.113347	0.133421	13.6916	7.1917
900	0.700456	0.071449	−0.008998	0.144918	0.217609	17.3622	8.1455
1350	0.694031	0.097213	−0.011839	0.170923	0.296059	20.3467	9.1301
1800	0.687614	0.121856	−0.014435	0.193427	0.371114	22.8982	10.1824
2250	0.681868	0.145726	−0.016831	0.213512	0.443824	25.1503	11.3355
2700	0.676800	0.169024	−0.019061	0.231809	0.514784	27.1811	12.6247
3150	0.672223	0.192024	−0.021162	0.248836	0.584834	29.0532	14.1034
3600	0.668317	0.214361	−0.023111	0.264520	0.652853	30.7627	15.7924
4050	0.664666	0.237050	−0.025005	0.279729	0.721934	32.4068	17.8481
4500	0.661475	0.258481	−0.026722	0.293520	0.787180	33.8861	20.2159
4950	0.658547	0.279835	−0.028368	0.307513	0.852174	35.2982	23.1471
5400	0.655796	0.301717	−0.029986	0.319869	0.918776	36.6828	26.9834
5850	0.653334	0.323022	−0.031493	0.332162	0.983596	37.9729	31.9113
6300	0.651017	0.344377	−0.032955	0.344159	1.048570	39.2247	38.7840
6750	0.648819	0.365337	−0.034365	0.355774	1.112348	40.4330	48.9625

**Table A6:** The numerical calculation results of the undetermined constants *β*, *c*_0_, *c*_1_, and *d*_0_ as well as maximum stress *σ_m_* and maximum deflection *w_m_* when *k* = 0.5 N/mm, *a* = 70 mm, *h* = 0.1 mm, *t* = 0.1 mm, *E* = 3.01 MPa, *ν* = 0.45, *g* = 5 mm, Δ*l* = 5 mm, *L* = 40 mm, and the wind pressure *q* takes different values.

*q*/Pa	*β*	*c*_0_	*c_1_*	*d*_0_	*σ_m_/*MPa	*w_m_*/mm	*C*/pF
54.5	0.504683	0.023909	−0.004348	0.109770	0.075129	10.00015	3.8922
500	0.732368	0.079161	−0.008883	0.143855	0.240433	13.3247	4.3002
1000	0.735016	0.131186	−0.013015	0.184199	0.397997	16.8019	4.8298
1500	0.729675	0.181754	−0.016657	0.219000	0.551757	19.7444	5.3916
2000	0.723679	0.231645	−0.019927	0.250047	0.702417	22.3230	6.0037
2500	0.716990	0.279793	−0.022831	0.277421	0.848754	24.5634	6.6607
3000	0.713010	0.330253	−0.025653	0.304747	1.001299	26.7632	7.4625
3500	0.708746	0.378837	−0.028191	0.329421	1.148490	28.7249	8.3600
4000	0.704376	0.427835	−0.030597	0.352975	1.297116	30.5757	9.4299
4500	0.700561	0.476959	−0.032877	0.375651	1.446090	32.4324	10.8189
5000	0.697314	0.524673	−0.034982	0.396920	1.590723	33.9729	12.3253
5500	0.694342	0.571729	−0.036966	0.417242	1.733400	35.5213	14.3309
6000	0.691476	0.620256	−0.038923	0.437581	1.880550	37.0572	17.0892
6500	0.688819	0.668647	−0.040792	0.457298	2.027310	38.5329	20.9664
7000	0.686385	0.716643	−0.042571	0.476358	2.172881	39.9471	26.7918

**Table A7:** The numerical calculation results of the undetermined constants *β*, *c*_0_, *c*_1_, and *d*_0_ as well as maximum stress *σ_m_* and maximum deflection *w_m_* when *k* = 0.5 N/mm, *a* = 70 mm, *h* = 0.5 mm, *t* = 0.1 mm, *E* = 3.01 MPa, *ν* = 0.45, *g* = 5 mm, Δ*l* = 5 mm, *L* = 40 mm, and the wind pressure *q* takes different values.

*q*/Pa	*β*	*c*_0_	*c_1_*	*d*_0_	*σ_m_/*MPa	*w_m_*/mm	*C*/pF
272	0.500873	0.023896	−0.004311	0.110235	0.075064	10.00003	3.8921
1600	0.650959	0.069444	−0.010107	0.154929	0.212687	14.4903	4.4643
3200	0.647272	0.115047	−0.015574	0.198540	0.352011	18.4655	5.1321
4800	0.640592	0.157020	−0.020196	0.236404	0.480293	21.6843	5.8395
6400	0.634700	0.196961	−0.024222	0.266922	0.602320	24.3005	6.5763
8000	0.629711	0.235558	−0.027849	0.293877	0.720184	26.5760	7.3868
9600	0.625830	0.274003	−0.031096	0.319211	0.837682	28.6803	8.3372
11200	0.622372	0.311102	−0.034047	0.341872	0.950778	30.5440	9.4093
12800	0.618950	0.345548	−0.036603	0.361610	1.061940	32.1508	10.5825
14400	0.615850	0.381522	−0.039106	0.381203	1.171510	33.7294	12.0599
16000	0.613148	0.417107	−0.041442	0.399901	1.273850	35.2211	13.8926
17600	0.610823	0.452363	−0.043631	0.417891	1.381160	36.6431	16.2461
19200	0.608781	0.487299	−0.045680	0.435156	1.487460	37.9953	19.3658
20800	0.606930	0.521925	−0.047596	0.451683	1.592850	39.2782	23.6799
22400	0.605195	0.556306	−0.049407	0.467611	1.697530	40.5057	30.0945

**Table A8:** The numerical calculation results of the undetermined constants *β*, *c*_0_, *c*_1_, and *d*_0_ as well as maximum stress *σ_m_* and maximum deflection *w_m_* when *k* = 0.5 N/mm, *a* = 70 mm, *h* = 0.3 mm, *t* = 2.5 mm, *E* = 3.01 MPa, *ν* = 0.45, *g* = 5 mm, Δ*l* = 5 mm, *L* = 40 mm, and the wind pressure *q* takes different values.

*q*/Pa	*β*	*c*_0_	*c_1_*	*d*_0_	*σ_m_/*MPa	*w_m_*/mm	*C/*pF
164	0.508089	0.023938	−0.004382	0.109411	0.075231	10.00132	3.7959
180	0.544050	0.024999	−0.004715	0.107564	0.078310	10.0430	3.8003
250	0.598700	0.029582	−0.005228	0.109212	0.091588	10.2999	3.8277
450	0.649548	0.040977	−0.006322	0.119276	0.125661	11.2394	3.9314
650	0.666143	0.051272	−0.007420	0.129987	0.156822	12.2096	4.0445
1000	0.675506	0.068129	−0.009289	0.147594	0.208037	13.7933	4.2439
2000	0.674910	0.112451	−0.014101	0.189765	0.342976	17.5352	4.8032
3000	0.668757	0.153851	−0.018268	0.224077	0.469091	20.5216	5.3678
4000	0.662773	0.193468	−0.021953	0.253639	0.590176	23.0514	5.9615
5000	0.657493	0.232254	−0.025264	0.279991	0.707882	25.2732	6.6028
6000	0.653780	0.269163	−0.028209	0.304693	0.825293	27.3276	7.3321
7000	0.649841	0.307092	−0.031039	0.326136	0.939422	29.0912	8.1002
8000	0.645980	0.344476	−0.033645	0.347378	1.051860	30.8186	9.0264
9000	0.642338	0.381389	−0.036051	0.367314	1.156710	32.4215	10.0977
10000	0.639188	0.416372	−0.038191	0.385375	1.268142	33.8582	11.2998
11000	0.636385	0.452081	−0.040249	0.403105	1.375390	35.2552	12.7791
12000	0.633941	0.488016	−0.042216	0.420358	1.483440	36.6031	14.6267
13000	0.631907	0.521772	−0.043989	0.436153	1.590910	37.8286	16.8402
14000	0.629948	0.557385	−0.045807	0.452525	1.697980	39.0928	19.9556
15000	0.628117	0.592392	−0.047568	0.468470	1.803200	40.3209	24.3276

**Table A9:** The numerical calculation results of the undetermined constants *β*, *c*_0_, *c*_1_, and *d*_0_ as well as maximum stress *σ_m_* and maximum deflection *w_m_* when *k* = 0.5 N/mm, *a* = 70 mm, *h* = 0.3 mm, *t* = 5 mm, *E* = 3.01 MPa, *ν* = 0.45, *g* = 5 mm, Δ*l* = 5 mm, *L* = 40 mm, and the wind pressure *q* takes different values.

*q*/Pa	*β*	*c*_0_	*c_1_*	*d*_0_	*σ_m_/*MPa	*w_m_*/mm	*C/*pF
164	0.508089	0.023938	−0.004382	0.109411	0.075231	10.00132	3.7005
180	0.544050	0.024999	−0.004715	0.107564	0.078310	10.0430	3.7047
250	0.598700	0.029582	−0.005228	0.109212	0.091588	10.2999	3.7307
450	0.649548	0.040977	−0.006322	0.119276	0.125661	11.2394	3.8291
650	0.666143	0.051272	−0.007420	0.129987	0.156822	12.2096	3.9364
1000	0.675506	0.068129	−0.009289	0.147594	0.208037	13.7933	4.1250
2000	0.674910	0.112451	−0.014101	0.189765	0.342976	17.5352	4.6514
3000	0.668757	0.153851	−0.018268	0.224077	0.469091	20.5216	5.1790
4000	0.662773	0.193468	−0.021953	0.253639	0.590176	23.0514	5.7294
5000	0.657493	0.232254	−0.025264	0.279991	0.707882	25.2732	6.3193
6000	0.653780	0.269163	−0.028209	0.304693	0.825293	27.3276	6.9842
7000	0.649841	0.307092	−0.031039	0.326136	0.939422	29.0912	7.6777
8000	0.645980	0.344476	−0.033645	0.347378	1.051860	30.8186	8.5049
9000	0.642338	0.381389	−0.036051	0.367314	1.156710	32.4215	9.4495
10000	0.639188	0.416372	−0.038191	0.385375	1.268142	33.8582	10.4943
11000	0.636385	0.452081	−0.040249	0.403105	1.375390	35.2552	11.7583
12000	0.633941	0.488016	−0.042216	0.420358	1.483440	36.6031	13.3046
13000	0.631907	0.521772	−0.043989	0.436153	1.590910	37.8286	15.1114
14000	0.629948	0.557385	−0.045807	0.452525	1.697980	39.0928	17.5732
15000	0.628117	0.592392	−0.047568	0.468470	1.803200	40.3209	20.8773

**Table A10:** The numerical calculation results of the undetermined constants *β*, *c*_0_, *c*_1_, and *d*_0_ as well as maximum stress *σ_m_* and maximum deflection *w_m_* when *k* = 0.5 N/mm, *a* = 70 mm, *h* = 0.3 mm, *t* = 0.1 mm, *E* = 5 MPa, *ν* = 0.45, *g* = 5 mm, Δ*l* = 5 mm, *L* = 40 mm, and the wind pressure *q* takes different values.

*q*/Pa	*β*	*c*_0_	*c_1_*	*d*_0_	*σ_m_/*MPa	*w_m_*/mm	*C*/pF
272	0.506278	0.023924	−0.004365	0.109603	0.124889	10.00132	3.8923
1600	0.651132	0.069586	−0.010120	0.155046	0.354013	14.5006	4.4658
3200	0.647404	0.115300	−0.015596	0.200633	0.586010	18.5626	5.1510
4800	0.640709	0.157381	−0.020224	0.236633	0.799644	21.7036	5.8444
6400	0.634806	0.197428	−0.024255	0.267195	1.002880	24.3231	6.5834
8000	0.629020	0.236678	−0.027884	0.294250	1.202070	26.6096	7.4003
9600	0.625553	0.273860	−0.031062	0.319114	1.390482	28.6365	8.3149
11200	0.621988	0.311804	−0.034075	0.340597	1.582810	30.5410	9.4073
12800	0.618661	0.347240	−0.036700	0.362150	1.762241	32.1954	10.6193
14400	0.615616	0.382484	−0.039152	0.382598	1.940730	33.7548	12.0870
16000	0.613236	0.418187	−0.041615	0.401429	2.121400	35.3408	14.0641
17600	0.610975	0.453787	−0.043676	0.418558	2.301466	36.6933	16.3438
19200	0.608904	0.488583	−0.045842	0.435712	2.477470	38.0371	19.4814
20800	0.606978	0.523341	−0.047662	0.452295	2.653190	39.3255	23.8760
22400	0.605232	0.557826	−0.049476	0.468252	2.827480	40.5548	30.4242

**Table A11:** The numerical calculation results of the undetermined constants *β*, *c*_0_, *c*_1_, and *d*_0_ as well as maximum stress *σ_m_* and maximum deflection *w_m_* when *k* = 0.5 N/mm, *a* = 70 mm, *h* = 0.3 mm, *t* = 0.1 mm, *E* = 7.84 MPa, *ν* = 0.45, *g* = 5 mm, Δ*l* = 5 mm, *L* = 40 mm, and the wind pressure *q* takes different values.

*q*/Pa	*β*	*c*_0_	*c_1_*	*d*_0_	*σ_m_/*MPa	*w_m_*/mm	*C*/pF
425.5	0.503027	0.023904	−0.004332	0.109969	0.195615	10.00048	3.8922
2200	0.629902	0.065748	−0.010172	0.155656	0.526224	14.5719	4.4762
4400	0.626162	0.108487	−0.015720	0.200883	0.867405	18.6248	5.1631
6600	0.620013	0.147354	−0.020357	0.236029	1.177759	21.7067	5.8451
8800	0.616775	0.185102	−0.024487	0.267615	1.462990	24.4164	6.6132
11000	0.610230	0.219360	−0.027927	0.291563	1.752221	26.4598	7.3406
13200	0.606452	0.253633	−0.031122	0.314971	2.025360	28.4212	8.2072
15400	0.603421	0.286574	−0.033982	0.336210	2.287700	30.2740	9.2372
17600	0.600372	0.320005	−0.036692	0.356434	2.553775	31.8362	10.3303
19800	0.597814	0.352086	−0.039132	0.374988	2.808960	33.3409	11.6593
22000	0.595601	0.384164	−0.041430	0.392831	3.063970	34.7744	13.2879
24200	0.593635	0.415905	−0.043579	0.409848	3.316183	36.1295	15.3094
26400	0.591816	0.447284	−0.045595	0.426102	3.565410	37.4137	17.8883
28600	0.590153	0.478166	−0.047473	0.441568	3.810578	38.6259	21.2706
30800	0.588624	0.509222	−0.049262	0.456646	4.057020	39.7981	26.0298

**Table A12:** The numerical calculation results of the undetermined constants *β*, *c*_0_, *c*_1_, and *d*_0_ as well as maximum stress *σ_m_* and maximum deflection *w_m_* when *k* = 0.5 N/mm, *a* = 70 mm, *h* = 0.3 mm, *t* = 0.1 mm, *E* = 3.01 MPa, *ν* = 0.15, *g* = 5 mm, Δ*l* = 5 mm, *L* = 40 mm, and the wind pressure *q* takes different values.

*q*/Pa	*β*	*c*_0_	*c_1_*	*d*_0_	*σ_m_/*MPa	*w_m_*/mm	*C*/pF
114	0.502118	0.017071	−0.004091	0.111054	0.054335	10.00006	3.8921
800	0.696036	0.050333	−0.009000	0.148883	0.154106	13.7861	4.3637
1600	0.695694	0.083397	−0.013576	0.191910	0.254962	17.5459	4.9605
2400	0.689363	0.114742	−0.017549	0.227568	0.350638	20.5983	5.5800
3200	0.683099	0.145103	−0.021077	0.258646	0.443319	23.2117	6.2482
4000	0.676066	0.174679	−0.024248	0.286202	0.533598	25.4956	6.9784
4800	0.672640	0.203992	−0.027167	0.312231	0.623050	27.6181	7.8288
5600	0.668516	0.233131	−0.029878	0.336452	0.711961	29.5698	8.8166
6400	0.664515	0.261286	−0.032336	0.358562	0.797859	31.3318	9.9501
7200	0.660951	0.290251	−0.034721	0.380394	0.886219	33.0526	11.3788
8000	0.658013	0.317497	−0.036847	0.400205	0.969318	34.5988	13.0643
8800	0.655111	0.344901	−0.038884	0.419481	1.052890	36.0901	15.2419
9600	0.652655	0.372902	−0.040861	0.438531	1.138277	37.5505	18.2152
10400	0.650328	0.400352	−0.042700	0.456624	1.221970	38.9247	22.3104
11200	0.648163	0.427854	−0.044472	0.474342	1.305820	40.2608	28.5514

**Table A13:** The numerical calculation results of the undetermined constants *β*, *c*_0_, *c*_1_, and *d*_0_ as well as maximum stress *σ_m_* and maximum deflection *w_m_* when *k* = 0.5 N/mm, *a* = 70 mm, *h* = 0.3 mm, *t* = 0.1 mm, *E* = 3.01 MPa, *ν* = 0.3, *g* = 5 mm, Δ*l* = 5 mm, *L* = 40 mm, and the wind pressure *q* takes different values.

*q*/Pa	*β*	*c*_0_	*c_1_*	*d*_0_	*σ_m_/*MPa	*w_m_*/mm	*C*/pF
133.5	0.502762	0.019770	−0.004206	0.110515	0.062554	10.00013	3.8922
900	0.687098	0.058331	−0.009240	0.149294	0.178366	13.8799	4.3768
1800	0.686150	0.096640	−0.014001	0.192514	0.295120	17.6813	4.9850
2700	0.679709	0.132723	−0.018120	0.227982	0.405157	20.7384	5.6122
3600	0.673471	0.167542	−0.021766	0.258715	0.511337	23.3412	6.2855
4500	0.667132	0.201523	−0.025047	0.285957	0.614946	25.6157	7.0216
5400	0.663170	0.234853	−0.028031	0.311402	0.716535	27.7057	7.8683
6300	0.659129	0.268199	−0.030816	0.335195	0.818157	29.6372	8.8552
7200	0.655226	0.300167	−0.033318	0.356708	0.915562	31.3648	9.9741
8100	0.651690	0.33323	−0.035754	0.378028	1.016280	33.0586	11.3845
9000	0.648877	0.365298	−0.037984	0.397942	1.108540	34.6246	13.0967
9900	0.646172	0.395755	−0.039996	0.416227	1.206697	36.0496	15.1733
10800	0.643710	0.426985	−0.041964	0.434404	1.301780	37.4549	17.9855
11700	0.641407	0.458348	−0.043843	0.452102	1.397261	38.8114	21.9044
12600	0.639304	0.489624	−0.045625	0.469250	1.492460	40.1146	27.7034

**Table A14:** The numerical calculation results of the undetermined constants *β*, *c*_0_, *c*_1_, and *d*_0_ as well as maximum stress *σ_m_* and maximum deflection *w_m_* when *k* = 0.5 N/mm, *a* = 70 mm, *h* = 0.3 mm, *t* = 0.1 mm, *E* = 3.01 MPa, *ν* = 0.45, *g* = 1 mm, Δ*l* = 5 mm, *L* = 40 mm, and the wind pressure *q* takes different values.

*q*/Pa	*β*	*c*_0_	*c_1_*	*d*_0_	*σ_m_/*MPa	*w_m_*/mm	*C*/pF
35.6	0.507215	0.008550	−0.001603	0.065493	0.026904	6.00023	3.8922
850	0.724312	0.053934	−0.006581	0.120561	0.164006	11.2669	4.5807
1700	0.708062	0.092562	−0.010893	0.162745	0.281578	15.0630	5.2502
2550	0.695527	0.128630	−0.014666	0.196068	0.391411	18.0058	5.9210
3400	0.685891	0.163203	−0.018040	0.224411	0.496698	20.4687	6.6300
4250	0.678180	0.196765	−0.021100	0.249483	0.598896	22.6166	7.4031
5100	0.671805	0.229586	−0.023904	0.272209	0.698823	24.5386	8.2656
5950	0.666403	0.261833	−0.026494	0.293152	0.796988	26.2896	9.2469
6800	0.661735	0.293621	−0.028903	0.312689	0.893734	27.9056	10.3849
7650	0.657642	0.325028	−0.031156	0.331081	0.989303	29.4120	11.7306
8500	0.654006	0.356114	−0.033271	0.348522	1.083876	30.8274	13.3569
9350	0.650745	0.386922	−0.035266	0.365158	1.177589	32.1659	15.3722
10200	0.647795	0.417487	−0.037154	0.381102	1.270550	33.4384	17.9465
11050	0.645107	0.447839	−0.038947	0.396444	1.362843	34.6534	21.3622
11900	0.642643	0.477998	−0.040653	0.411257	1.454539	35.8182	26.1300

**Table A15:** The numerical calculation results of the undetermined constants *β*, *c*_0_, *c*_1_, and *d*_0_ as well as maximum stress *σ_m_* and maximum deflection *w_m_* when *k* = 0.5 N/mm, *a* = 70 mm, *h* = 0.3 mm, *t* = 0.1 mm, *E* = 3.01 MPa, *ν* = 0.45, *g* = 3 mm, Δ*l* = 5 mm, *L* = 40 mm, and the wind pressure *q* takes different values.

*q*/Pa	*β*	*c*_0_	*c_1_*	*d*_0_	*σ_m_/*MPa	*w_m_*/mm	*C*/pF
84.3	0.508782	0.015260	−0.002837	0.087317	0.047992	8.00080	3.8922
900	0.699680	0.059863	−0.007746	0.132820	0.182392	12.4192	4.4539
1800	0.691609	0.100386	−0.012240	0.174406	0.305766	16.1385	5.0698
2700	0.682432	0.138220	−0.016151	0.207762	0.421005	19.0663	5.6890
3600	0.674713	0.174507	−0.019633	0.236324	0.531534	21.5328	6.3415
4500	0.668274	0.209756	−0.022779	0.261691	0.638880	23.6921	7.0493
5400	0.662819	0.244244	−0.025653	0.284747	0.743890	25.6294	7.8339
6300	0.658120	0.278145	−0.028301	0.306040	0.847089	27.3977	8.7196
7200	0.654013	0.311575	−0.030758	0.325936	0.948830	29.0322	9.7373
8100	0.650379	0.344616	−0.033051	0.344693	1.049364	30.5579	10.9277
9000	0.647131	0.377326	−0.035202	0.362502	1.148873	31.9930	12.3477
9900	0.644200	0.409752	−0.037227	0.379507	1.247498	33.3514	14.0795
10800	0.641537	0.441929	−0.039140	0.395820	1.345349	34.6440	16.2478
11700	0.639101	0.473886	−0.040955	0.411532	1.442513	35.8793	19.0519
12600	0.636860	0.505646	−0.042681	0.426714	1.539062	37.0644	22.8322
13500	0.634789	0.537227	−0.044326	0.441426	1.635056	38.2048	28.2206

**Table A16:** The numerical calculation results of the undetermined constants *β*, *c*_0_, *c*_1_, and *d*_0_ as well as maximum stress *σ_m_* and maximum deflection *w_m_* when *k* = 0.5 N/mm, *a* = 70 mm, *h* = 0.3 mm, *t* = 0.1 mm, *E* = 3.01 MPa, *ν* = 0.45, *g* = 5 mm, Δ*l* = 1 mm, *L* = 40 mm, and the wind pressure *q* takes different values.

*q*/Pa	*β*	*c*_0_	*c_1_*	*d*_0_	*σ_m_/*MPa	*w_m_*/mm	*C*/pF
35.5	0.503347	0.008542	−0.001590	0.065756	0.026872	6.00005	3.4933
1020	0.720888	0.061962	−0.007496	0.130066	0.188433	12.1288	4.1439
2040	0.702618	0.107215	−0.012458	0.176841	0.326198	16.3139	4.7477
3060	0.689470	0.149516	−0.016732	0.213537	0.455015	19.5282	5.3459
4080	0.679600	0.190118	−0.020510	0.244680	0.578659	22.2073	5.9733
5100	0.671805	0.229586	−0.023904	0.272209	0.698823	24.5386	6.6526
6120	0.665416	0.268225	−0.026990	0.297162	0.816442	26.6227	7.4056
7140	0.660036	0.306226	−0.029822	0.320171	0.932091	28.5202	8.2564
8160	0.655411	0.343715	−0.032440	0.341650	1.046157	30.2712	9.2354
9180	0.651371	0.380781	−0.034876	0.361890	1.158910	31.9038	10.3835
10200	0.647795	0.417487	−0.037154	0.381102	1.270550	33.4384	11.7573
11220	0.644598	0.453885	−0.039294	0.399446	1.381228	34.8902	13.4395
12240	0.641712	0.490012	−0.041313	0.417047	1.491063	36.2713	15.5570
13260	0.639089	0.525898	−0.043223	0.434003	1.600150	37.5910	18.3143
14280	0.636688	0.561570	−0.045036	0.450393	1.708566	38.8569	22.0656
15300	0.634479	0.597047	−0.046763	0.466282	1.816376	40.0753	27.4840

**Table A17:** The numerical calculation results of the undetermined constants *β*, *c*_0_, *c*_1_, and *d*_0_ as well as maximum stress *σ_m_* and maximum deflection *w_m_* when *k* = 0.5 N/mm, *a* = 70 mm, *h* = 0.3 mm, *t* = 0.1 mm, *E* = 3.01 MPa, *ν* = 0.45, *g* = 5 mm, Δ*l* = 3 mm, *L* = 40 mm, and the wind pressure *q* takes different values.

*q*/Pa	*β*	*c*_0_	*c_1_*	*d*_0_	*σ_m_/*MPa	*w_m_*/mm	*C*/pF
84	0.504741	0.015240	−0.002812	0.087662	0.047920	8.00023	3.6820
1000	0.699278	0.064572	−0.008281	0.138046	0.196719	12.8905	4.2421
2000	0.689464	0.108968	−0.013153	0.182361	0.331905	16.8413	4.8365
3000	0.679697	0.150455	−0.017354	0.217714	0.458275	19.9300	5.4315
4000	0.671712	0.190282	−0.021068	0.247929	0.579576	22.5243	6.0574
5000	0.665138	0.228997	−0.024406	0.274744	0.697468	24.7919	6.7359
6000	0.659613	0.266902	−0.027441	0.299113	0.812866	26.8246	7.4877
7000	0.654881	0.304183	−0.030227	0.321621	0.926333	28.6791	8.3366
8000	0.650763	0.340962	−0.032804	0.342659	1.038247	30.3931	9.3123
9000	0.647131	0.377326	−0.035202	0.362502	1.148873	31.9930	10.4545
10000	0.643892	0.413339	−0.037444	0.381352	1.258407	33.4981	11.8181
11000	0.640977	0.449049	−0.039552	0.399361	1.366998	34.9232	13.4834
12000	0.638334	0.484494	−0.041540	0.416648	1.474761	36.2796	15.5718
13000	0.635920	0.519703	−0.043421	0.433307	1.581792	37.5764	18.2784
14000	0.633703	0.554702	−0.045208	0.449416	1.688164	38.8209	21.9378
14500	0.632660	0.572128	−0.046068	0.457283	1.741123	39.4254	24.3011
